# Supplementary material for: Association between confirmed congenital Zika infection at birth and outcomes up to 3 years of life
Source: Nat Commun. 2021 Jun 1;12:3270. doi: 10.1038/s41467-021-23468-3 (PMC8169933; doi:10.1038/s41467-021-23468-3)
Supplement: Supplementary file 1 — Supplementary Codes [file 41467_2021_23468_MOESM1_ESM.pdf]

```
------------------------------------------------------------------------------------------------------------------------------------
----------------------------------
      name:  <unnamed>
       log:  \\HOME\lpomar\Mes Documents\Zika_enfants-Guyane\Nature\R1\Dataset\Table1.log
  log type:  text
 opened on:  23 Mar 2021, 11:49:29

.
. ** Table 1: Baseline characteristics
. import excel "\\HOME\lpomar\Mes Documents\Zika_enfants-Guyane\Nature\R1\Dataset\Source Data.xlsx",
sheet("Table 1") firstrow
(24 vars, 132 obs)

.
. destring Laboratoryconfirm, replace
LaboratoryconfirmedCongenital: all characters numeric; replaced as byte
(3 missing values generated)

. tab Laboratoryconfirm

 Laboratory |
  confirmed |
 Congenital |
       ZIKA |
  infection |      Freq.     Percent        Cum.
------------+-----------------------------------
          0 |        111       86.05       86.05
          1 |         18       13.95      100.00
------------+-----------------------------------
      Total |        129      100.00

.
. * Maternal age:
. codebook Maternalage if Laboratoryconfirm==1

------------------------------------------------------------------------------------------------------------------------------------
----------------------------------
Maternalageatbirthyears                                                                                              Maternal age
at birth (years)
------------------------------------------------------------------------------------------------------------------------------------
----------------------------------

                  type:  numeric (byte)

                 range:  [18,38]                      units:  1
         unique values:  11                       missing .:  0/18

                  mean:   25.8889
              std. dev:   6.25807

           percentiles:        10%       25%       50%       75%       90%
                                19        20        25        30        38

. codebook Maternalage if Laboratoryconfirm==0

------------------------------------------------------------------------------------------------------------------------------------
----------------------------------
```

```
Maternalageatbirthyears                                                    Maternal age
at birth (years)
-----------------------------------------------------------------------------------------------
----------------------------------

                  type:  numeric (byte)

                 range:  [18,43]                     units:  1
         unique values:  21                      missing .:  0/111

                  mean:   26.5045
              std. dev:   6.06161

           percentiles:        10%       25%       50%       75%       90%
                                19        23        26        29        37

. ranksum Maternalage, by(Laboratoryconfirm)

Two-sample Wilcoxon rank-sum (Mann-Whitney) test

Laboratory~l |      obs    rank sum    expected
-------------+---------------------------------
           0 |      111        7295        7215
           1 |       18        1090        1170
-------------+---------------------------------
    combined |      129        8385        8385

unadjusted variance    21645.00
adjustment for ties     -150.83
                     ----------
adjusted variance      21494.17

Ho: Matern~s(Labora~l==0) = Matern~s(Labora~l==1)
             z =   0.546
    Prob > |z| =   0.5853
    Exact Prob =   0.5895

. stddiff Maternalage, by(Laboratoryconfirm)

-----------------------------------------------------------------------------
             |LaboratoryconfirmedCon~0 |LaboratoryconfirmedCon~1 |
             | Mean or N     SD or (%) | Mean or N     SD or (%) |  Std Diff
-------------+-------------------------+-------------------------+------------
Maternalag~s |      26.5        6.0616 |     25.89        6.2581 |    0.09993
-----------------------------------------------------------------------------


.
. * Maternal socio-economic status
. tab Socioeconomics if Laboratoryconfirm==1, freq

Socio-econo |
 mic status |      Freq.     Percent        Cum.
------------+-----------------------------------
          . |          3       16.67       16.67
          H |          1        5.56       22.22
          L |          6       33.33       55.56
          M |          8       44.44      100.00
```

```
------------+-----------------------------------
      Total |         18      100.00

. tab Socioeconomics if Laboratoryconfirm==0, freq

Socio-econo |
 mic status |      Freq.     Percent        Cum.
------------+-----------------------------------
          . |         19       17.12       17.12
          H |          8        7.21       24.32
          L |         38       34.23       58.56
          M |         46       41.44      100.00
------------+-----------------------------------
      Total |        111      100.00

. gen se_low=0

. replace se_low=1 if Socioeconomics=="L"
(45 real changes made)

. tab se_low Laboratoryconfirm, chi2

           | Laboratory confirmed
           |   Congenital ZIKA
           |      infection
    se_low |         0          1 |     Total
-----------+----------------------+----------
         0 |        73         12 |        85
         1 |        38          6 |        44
-----------+----------------------+----------
     Total |       111         18 |       129

          Pearson chi2(1) =   0.0056   Pr = 0.940

. stddiff se_low, by(Laboratoryconfirm)

-----------------------------------------------------------------------------
             |LaboratoryconfirmedCon~0 |LaboratoryconfirmedCon~1 |
             | Mean or N     SD or (%) | Mean or N     SD or (%) |  Std Diff
-------------+-------------------------+-------------------------+------------
      se low |     .3423       .47665 |     .3333       .48507 |    0.01873
-----------------------------------------------------------------------------


. gen se_mod=0

. replace se_mod=1 if Socioeconomics=="M"
(56 real changes made)

. tab se_mod Laboratoryconfirm, chi2

           | Laboratory confirmed
           |   Congenital ZIKA
           |      infection
    se_mod |         0          1 |     Total
-----------+----------------------+----------
         0 |        65         10 |        75
         1 |        46          8 |        54
```

```
-----------+----------------------+----------
     Total |       111         18 |       129

          Pearson chi2(1) =   0.0574   Pr = 0.811

. stddiff se_mod, by(Laboratoryconfirm)

--------------------------------------------------------------------------
             |LaboratoryconfirmedCon~0 |LaboratoryconfirmedCon~1 |
             | Mean or N     SD or (%) | Mean or N     SD or (%) |  Std Diff
-------------+------------------------+------------------------+------------
      se mod |     .4144       .49485 |     .4444       .51131 |   -0.05968
--------------------------------------------------------------------------

. gen se_hi=0

. replace se_hi=1 if Socioeconomics=="H"
(9 real changes made)

. tab se_hi Laboratoryconfirm, exact

           | Laboratory confirmed
           |    Congenital ZIKA
           |       infection
     se_hi |         0          1 |     Total
-----------+----------------------+----------
         0 |       103         17 |       120
         1 |         8          1 |         9
-----------+----------------------+----------
     Total |       111         18 |       129

           Fisher's exact =                 1.000
   1-sided Fisher's exact =                 0.634

. stddiff se_hi, by(Laboratoryconfirm)

--------------------------------------------------------------------------
             |LaboratoryconfirmedCon~0 |LaboratoryconfirmedCon~1 |
             | Mean or N     SD or (%) | Mean or N     SD or (%) |  Std Diff
-------------+------------------------+------------------------+------------
       se hi |    .07207       .25978 |    .05556        .2357 |    0.06659
--------------------------------------------------------------------------

. gen se_unk=0

. replace se_unk=1 if Socioeconomics=="."
(22 real changes made)

. tab se_unk Laboratoryconfirm, chi2

           | Laboratory confirmed
           |    Congenital ZIKA
           |       infection
    se_unk |         0          1 |     Total
-----------+----------------------+----------
         0 |        92         15 |       107
         1 |        19          3 |        22
```

```
-----------+----------------------+----------
     Total |       111         18 |       129 

          Pearson chi2(1) =   0.0022   Pr = 0.962

. stddiff se_unk, by(Laboratoryconfirm)

-----------------------------------------------------------------------------
             |LaboratoryconfirmedCon~0 |LaboratoryconfirmedCon~1 |
             | Mean or N     SD or (%) | Mean or N     SD or (%) |  Std Diff
-------------+-------------------------+-------------------------+------------
      se unk |     .1712        .37837 |     .1667        .38348 |    0.01182
-----------------------------------------------------------------------------


.
. * Maternal Exposures:
. tab Alcohol if Laboratoryconfirm==1, freq

    Alcohol |      Freq.     Percent        Cum.
------------+-----------------------------------
          0 |         17       94.44       94.44
          1 |          1        5.56      100.00
------------+-----------------------------------
      Total |         18      100.00

. tab Alcohol if Laboratoryconfirm==0, freq

    Alcohol |      Freq.     Percent        Cum.
------------+-----------------------------------
          0 |         99       89.19       89.19
          1 |         12       10.81      100.00
------------+-----------------------------------
      Total |        111      100.00

. tab Alcohol Laboratoryconfirm, exact

           | Laboratory confirmed
           |    Congenital ZIKA
           |       infection
   Alcohol |         0          1 |     Total
-----------+----------------------+----------
         0 |        99         17 |       116 
         1 |        12          1 |        13 
-----------+----------------------+----------
     Total |       111         18 |       129 

           Fisher's exact =                 0.692
   1-sided Fisher's exact =                 0.429

. stddiff Alcohol, by(Laboratoryconfirm)

-----------------------------------------------------------------------------
             |LaboratoryconfirmedCon~0 |LaboratoryconfirmedCon~1 |
             | Mean or N     SD or (%) | Mean or N     SD or (%) |  Std Diff
-------------+-------------------------+-------------------------+------------
     Alcohol |     .1081        .31193 |    .05556         .2357 |    0.19010
-----------------------------------------------------------------------------
```

```
.
. tab Drug if Laboratoryconfirm==1, freq

       Drug |      Freq.     Percent        Cum.
------------+-----------------------------------
          0 |         18      100.00      100.00
------------+-----------------------------------
      Total |         18      100.00

. tab Drug if Laboratoryconfirm==0, freq

       Drug |      Freq.     Percent        Cum.
------------+-----------------------------------
          0 |        110       99.10       99.10
          1 |          1        0.90      100.00
------------+-----------------------------------
      Total |        111      100.00

. tab Drug Laboratoryconfirm, exact

           | Laboratory confirmed
           |    Congenital ZIKA
           |       infection
      Drug |         0          1 |     Total
-----------+----------------------+----------
         0 |       110         18 |       128
         1 |         1          0 |         1
-----------+----------------------+----------
     Total |       111         18 |       129

           Fisher's exact =                 1.000
   1-sided Fisher's exact =                 0.860

. stddiff Drug, by(Laboratoryconfirm)

-------------------------------------------------------------------------
             |LaboratoryconfirmedCon~0 |LaboratoryconfirmedCon~1 |
             | Mean or N     SD or (%) | Mean or N     SD or (%) |  Std Diff
-------------+-------------------------+-------------------------+------------
        Drug |   .009009       .094916 |         0             0 |    0.13423
-------------------------------------------------------------------------


.
. tab Smoking if Laboratoryconfirm==1, freq

    Smoking |      Freq.     Percent        Cum.
------------+-----------------------------------
          0 |         17       94.44       94.44
          1 |          1        5.56      100.00
------------+-----------------------------------
      Total |         18      100.00

. tab Smoking if Laboratoryconfirm==0, freq

    Smoking |      Freq.     Percent        Cum.
------------+-----------------------------------
          0 |        105       95.45       95.45
```

```
         1 |          5        4.55      100.00
------------+-----------------------------------
      Total |        110      100.00

. tab Smoking Laboratoryconfirm, exact

           | Laboratory confirmed
           |    Congenital ZIKA
           |       infection
   Smoking |         0          1 |     Total
-----------+----------------------+----------
         0 |       105         17 |       122 
         1 |         5          1 |         6 
-----------+----------------------+----------
     Total |       110         18 |       128 

           Fisher's exact =                 1.000
   1-sided Fisher's exact =                 0.605

. stddiff Smoking, by(Laboratoryconfirm)

------------------------------------------------------------------------------
             |LaboratoryconfirmedCon~0 |LaboratoryconfirmedCon~1 |
             | Mean or N     SD or (%) | Mean or N     SD or (%) |  Std Diff
-------------+-------------------------+-------------------------+------------
     Smoking |    .04545        .20925 |    .05556         .2357 |   -0.04532
------------------------------------------------------------------------------


. 
. tab Leadpois if Laboratoryconfirm==1, freq

       Lead |
  poisoning |      Freq.     Percent        Cum.
------------+-----------------------------------
          0 |         17       94.44       94.44
          1 |          1        5.56      100.00
------------+-----------------------------------
      Total |         18      100.00

. tab Leadpois if Laboratoryconfirm==0, freq

       Lead |
  poisoning |      Freq.     Percent        Cum.
------------+-----------------------------------
          0 |        105       94.59       94.59
          1 |          6        5.41      100.00
------------+-----------------------------------
      Total |        111      100.00

. tab Leadpois Laboratoryconfirm, exact

           | Laboratory confirmed
           |    Congenital ZIKA
      Lead |       infection
 poisoning |         0          1 |     Total
-----------+----------------------+----------
         0 |       105         17 |       122 
```

```
         1 |         6          1 |          7 
-----------+----------------------+----------
     Total |       111         18 |       129 

           Fisher's exact =                 1.000
   1-sided Fisher's exact =                 0.660

. stddiff Leadpois, by(Laboratoryconfirm)

-------------------------------------------------------------------------
             |LaboratoryconfirmedCon~0 |LaboratoryconfirmedCon~1 |
             | Mean or N     SD or (%) | Mean or N     SD or (%) |  Std Diff
-------------+-------------------------+-------------------------+------------
Leadpoison~g |    .05405       .22715 |    .05556        .2357 |   -0.00649
-------------------------------------------------------------------------


. 
. * Maternal Comorbidities:
. tab Anymat if Laboratoryconfirm==1, freq

        Any |
   maternal |
comorbiditi |
         es |      Freq.     Percent        Cum.
------------+-----------------------------------
          0 |         15       83.33       83.33
          1 |          3       16.67      100.00
------------+-----------------------------------
      Total |         18      100.00

. tab Anymat if Laboratoryconfirm==0, freq

        Any |
   maternal |
comorbiditi |
         es |      Freq.     Percent        Cum.
------------+-----------------------------------
          0 |         96       86.49       86.49
          1 |         15       13.51      100.00
------------+-----------------------------------
      Total |        111      100.00

. tab Anymat Laboratoryconfirm, exact

       Any | Laboratory confirmed
  maternal |    Congenital ZIKA
comorbidit |       infection
       ies |         0          1 |     Total
-----------+----------------------+----------
         0 |        96         15 |       111 
         1 |        15          3 |        18 
-----------+----------------------+----------
     Total |       111         18 |       129 

           Fisher's exact =                 0.717
   1-sided Fisher's exact =                 0.477
```

```
. stddiff Anymat, by(Laboratoryconfirm)

----------------------------------------------------------------------------
             |LaboratoryconfirmedCon~0 |LaboratoryconfirmedCon~1 |
             | Mean or N     SD or (%) | Mean or N     SD or (%) |  Std Diff
-------------+-------------------------+-------------------------+------------
Anymaterna~s |     .1351        .34342 |     .1667        .38348 |  -0.08662
----------------------------------------------------------------------------


.
. tab Diabetes if Laboratoryconfirm==1, freq

   Diabetes |
  (previous |
         or |
gestational |
          ) |      Freq.     Percent        Cum.
------------+-----------------------------------
          0 |         17       94.44       94.44
          1 |          1        5.56      100.00
------------+-----------------------------------
      Total |         18      100.00

. tab Diabetes if Laboratoryconfirm==0, freq

   Diabetes |
  (previous |
         or |
gestational |
          ) |      Freq.     Percent        Cum.
------------+-----------------------------------
          0 |        104       93.69       93.69
          1 |          7        6.31      100.00
------------+-----------------------------------
      Total |        111      100.00

. tab Diabetes Laboratoryconfirm, exact

  Diabetes |
 (previous | Laboratory confirmed
        or |   Congenital ZIKA
gestationa |       infection
        l) |         0          1 |     Total
-----------+----------------------+----------
         0 |       104         17 |       121
         1 |         7          1 |         8
-----------+----------------------+----------
     Total |       111         18 |       129

           Fisher's exact =                 1.000
   1-sided Fisher's exact =                 0.691

. stddiff Diabetes, by(Laboratoryconfirm)

----------------------------------------------------------------------------
             |LaboratoryconfirmedCon~0 |LaboratoryconfirmedCon~1 |
             | Mean or N     SD or (%) | Mean or N     SD or (%) |  Std Diff
```

```
-------------+------------------------+------------------------+------------
Diabetespr~a |    .06306       .24418 |    .05556        .2357 |    0.03128
----------------------------------------------------------------------------


.
. tab Vascular if Laboratoryconfirm==1, freq

   Vascular |
pathologies |      Freq.     Percent        Cum.
------------+-----------------------------------
          0 |         17       94.44       94.44
          1 |          1        5.56      100.00
------------+-----------------------------------
      Total |         18      100.00

. tab Vascular if Laboratoryconfirm==0, freq

   Vascular |
pathologies |      Freq.     Percent        Cum.
------------+-----------------------------------
          0 |        106       95.50       95.50
          1 |          5        4.50      100.00
------------+-----------------------------------
      Total |        111      100.00

. tab Vascular Laboratoryconfirm, exact

           | Laboratory confirmed
  Vascular |   Congenital ZIKA
pathologie |       infection
         s |         0          1 |     Total
-----------+----------------------+----------
         0 |       106         17 |       123
         1 |         5          1 |         6
-----------+----------------------+----------
     Total |       111         18 |       129

           Fisher's exact =                 1.000
   1-sided Fisher's exact =                 0.602

. stddiff Vascular, by(Laboratoryconfirm)

----------------------------------------------------------------------------
             |LaboratoryconfirmedCon~0 |LaboratoryconfirmedCon~1 |
             | Mean or N     SD or (%) | Mean or N     SD or (%) |  Std Diff
-------------+------------------------+------------------------+------------
Vascularpa~s |    .04505       .20834 |    .05556        .2357 |   -0.04725
----------------------------------------------------------------------------


.
. tab Severeanemia if Laboratoryconfirm==1, freq

     Severe |
     anemia |      Freq.     Percent        Cum.
------------+-----------------------------------
          0 |         17       94.44       94.44
          1 |          1        5.56      100.00
```

```
------------+-----------------------------------
      Total |         18      100.00

. tab Severeanemia if Laboratoryconfirm==0, freq

     Severe |
     anemia |      Freq.     Percent        Cum.
------------+-----------------------------------
          0 |        106       95.50       95.50
          1 |          5        4.50      100.00
------------+-----------------------------------
      Total |        111      100.00

. tab Severeanemia Laboratoryconfirm, exact

           | Laboratory confirmed
           |    Congenital ZIKA
    Severe |       infection
    anemia |         0          1 |     Total
-----------+----------------------+----------
         0 |       106         17 |       123 
         1 |         5          1 |         6 
-----------+----------------------+----------
     Total |       111         18 |       129 

           Fisher's exact =                 1.000
   1-sided Fisher's exact =                 0.602

. stddiff Severeanemia, by(Laboratoryconfirm)

----------------------------------------------------------------------------
             |LaboratoryconfirmedCon~0 |LaboratoryconfirmedCon~1 |
             | Mean or N     SD or (%) | Mean or N     SD or (%) |  Std Diff
-------------+-------------------------+-------------------------+------------
Severeanemia |    .04505        .20834 |    .05556        .2357 |   -0.04725
----------------------------------------------------------------------------


. 
. tab Coinf if Laboratoryconfirm==1, freq

Co-infectio |
         ns |      Freq.     Percent        Cum.
------------+-----------------------------------
          0 |         17       94.44       94.44
          1 |          1        5.56      100.00
------------+-----------------------------------
      Total |         18      100.00

. tab Coinf if Laboratoryconfirm==0, freq

Co-infectio |
         ns |      Freq.     Percent        Cum.
------------+-----------------------------------
          0 |        109       98.20       98.20
          1 |          2        1.80      100.00
------------+-----------------------------------
      Total |        111      100.00
```

```
. tab Coinf Laboratoryconfirm, exact

           | Laboratory confirmed
           |    Congenital ZIKA
Co-infecti |       infection
      ons |         0          1 |     Total
-----------+----------------------+----------
         0 |       109         17 |       126
         1 |         2          1 |         3
-----------+----------------------+----------
     Total |       111         18 |       129

           Fisher's exact =                 0.365
   1-sided Fisher's exact =                 0.365

. stddiff Coinf, by(Laboratoryconfirm)

---------------------------------------------------------------------------
             |LaboratoryconfirmedCon~0 |LaboratoryconfirmedCon~1 |
             | Mean or N     SD or (%) | Mean or N     SD or (%) |  Std Diff
-------------+-------------------------+-------------------------+------------
Coinfections |    .01802       .13362 |    .05556        .2357 |   -0.19593
---------------------------------------------------------------------------


.
. * Maternal ZIKV infection:
. tab SymptomaticM if Laboratoryconfirm==1, freq

Symptomatic |
   Maternal |
       Zika |
 infection  |      Freq.     Percent        Cum.
------------+-----------------------------------
          0 |         14       77.78       77.78
          1 |          4       22.22      100.00
------------+-----------------------------------
      Total |         18      100.00

. tab SymptomaticM if Laboratoryconfirm==0, freq

Symptomatic |
   Maternal |
       Zika |
 infection  |      Freq.     Percent        Cum.
------------+-----------------------------------
          0 |         89       80.18       80.18
          1 |         22       19.82      100.00
------------+-----------------------------------
      Total |        111      100.00

. tab SymptomaticM Laboratoryconfirm, chi2

Symptomati | Laboratory confirmed
c Maternal |    Congenital ZIKA
      Zika |       infection
infection  |         0          1 |     Total
-----------+----------------------+----------
```

```
         0 |        89         14 |       103 
         1 |        22          4 |        26 
-----------+----------------------+----------
     Total |       111         18 |       129 

          Pearson chi2(1) =   0.0555   Pr = 0.814

. stddiff SymptomaticM, by(Laboratoryconfirm)

---------------------------------------------------------------------------
             |LaboratoryconfirmedCon~0 |LaboratoryconfirmedCon~1 |
             | Mean or N     SD or (%) | Mean or N     SD or (%) |  Std Diff
-------------+-------------------------+-------------------------+------------
Symptomati~t |     .1982        .40045 |     .2222        .42779 |   -0.05798
---------------------------------------------------------------------------


.
. tab Trimester if Laboratoryconfirm==1, freq

  Trimester |
of Maternal |
       Zika |
  infection |      Freq.     Percent        Cum.
------------+-----------------------------------
          . |          2       11.11       11.11
          1 |          7       38.89       50.00
          2 |          7       38.89       88.89
          3 |          2       11.11      100.00
------------+-----------------------------------
      Total |         18      100.00

. tab Trimester if Laboratoryconfirm==0, freq

  Trimester |
of Maternal |
       Zika |
  infection |      Freq.     Percent        Cum.
------------+-----------------------------------
          . |         22       19.82       19.82
          1 |         27       24.32       44.14
          2 |         37       33.33       77.48
          3 |         25       22.52      100.00
------------+-----------------------------------
      Total |        111      100.00

. gen t1=0

. replace t1=1 if Trimester=="1"
(34 real changes made)

. tab t1 Laboratoryconfirm, chi2

           | Laboratory confirmed
           |   Congenital ZIKA
           |      infection
        t1 |         0          1 |     Total
-----------+----------------------+----------
```

```
         0 |        84         11 |         95 
         1 |        27          7 |         34 
-----------+----------------------+----------
     Total |       111         18 |        129 

          Pearson chi2(1) =   1.6927   Pr = 0.193

. stddiff t1, by(Laboratoryconfirm)

--------------------------------------------------------------------------
             |LaboratoryconfirmedCon~0 |LaboratoryconfirmedCon~1 |
             | Mean or N     SD or (%) | Mean or N     SD or (%) |  Std Diff
-------------+-------------------------+-------------------------+------------
          t1 |     .2432       .43099 |     .3889       .50163 |   -0.31145
--------------------------------------------------------------------------

. gen t2=0

. replace t2=1 if Trimester=="2"
(45 real changes made)

. tab t2 Laboratoryconfirm, chi2

           | Laboratory confirmed
           |    Congenital ZIKA
           |       infection
        t2 |         0          1 |     Total
-----------+----------------------+----------
         0 |        74         11 |         85 
         1 |        37          7 |         44 
-----------+----------------------+----------
     Total |       111         18 |        129 

          Pearson chi2(1) =   0.2127   Pr = 0.645

. stddiff t2, by(Laboratoryconfirm)

--------------------------------------------------------------------------
             |LaboratoryconfirmedCon~0 |LaboratoryconfirmedCon~1 |
             | Mean or N     SD or (%) | Mean or N     SD or (%) |  Std Diff
-------------+-------------------------+-------------------------+------------
          t2 |     .3333       .47354 |     .3889       .50163 |   -0.11389
--------------------------------------------------------------------------

. gen t3=0

. replace t3=1 if Trimester=="3"
(29 real changes made)

. tab t3 Laboratoryconfirm, exact

           | Laboratory confirmed
           |    Congenital ZIKA
           |       infection
        t3 |         0          1 |     Total
-----------+----------------------+----------
         0 |        86         16 |        102 
```

```
        1 |        25          2 |        27 
-----------+----------------------+----------
     Total |       111         18 |       129 

           Fisher's exact =                 0.360
   1-sided Fisher's exact =                 0.220

. stddiff t3, by(Laboratoryconfirm)

--------------------------------------------------------------------------
             |LaboratoryconfirmedCon~0 |LaboratoryconfirmedCon~1 |
             | Mean or N     SD or (%) | Mean or N     SD or (%) |  Std Diff
-------------+-------------------------+-------------------------+------------
          t3 |     .2252        .41963 |     .1111        .32338 |    0.30462
--------------------------------------------------------------------------

. gen t_unk=0

. replace t_unk=1 if Trimester=="."
(24 real changes made)

. tab t_unk Laboratoryconfirm, exact

           | Laboratory confirmed
           |    Congenital ZIKA
           |       infection
     t_unk |         0          1 |     Total
-----------+----------------------+----------
         0 |        89         16 |       105 
         1 |        22          2 |        24 
-----------+----------------------+----------
     Total |       111         18 |       129 

           Fisher's exact =                 0.523
   1-sided Fisher's exact =                 0.304

. stddiff t_unk, by(Laboratoryconfirm)

--------------------------------------------------------------------------
             |LaboratoryconfirmedCon~0 |LaboratoryconfirmedCon~1 |
             | Mean or N     SD or (%) | Mean or N     SD or (%) |  Std Diff
-------------+-------------------------+-------------------------+------------
       t unk |     .1982        .40045 |     .1111        .32338 |    0.23928
--------------------------------------------------------------------------


. 
. * Dichorionic twins
. tab Dichorionictwins if Laboratoryconfirm==1, freq

Dichorionic |
     twins  |      Freq.     Percent        Cum.
------------+-----------------------------------
          0 |         16       88.89       88.89
          1 |          2       11.11      100.00
------------+-----------------------------------
      Total |         18      100.00
```

```
. tab Dichorionictwins if Laboratoryconfirm==0, freq

Dichorionic |
      twins |      Freq.     Percent        Cum.
------------+-----------------------------------
          0 |        105       94.59       94.59
          1 |          6        5.41      100.00
------------+-----------------------------------
      Total |        111      100.00

. tab Dichorionictwins Laboratoryconfirm, exact

           | Laboratory confirmed
           |    Congenital ZIKA
Dichorioni |       infection
  c twins  |         0          1 |     Total
-----------+----------------------+----------
         0 |       105         16 |       121
         1 |         6          2 |         8
-----------+----------------------+----------
     Total |       111         18 |       129

           Fisher's exact =                 0.309
   1-sided Fisher's exact =                 0.309

. stddiff Dichorionictwins, by(Laboratoryconfirm)

------------------------------------------------------------------------------
             |LaboratoryconfirmedCon~0 |LaboratoryconfirmedCon~1 |
             | Mean or N     SD or (%) | Mean or N     SD or (%) |  Std Diff
-------------+-------------------------+-------------------------+------------
Dichorioni~s |    .05405        .22715 |     .1111        .32338 |   -0.20418
------------------------------------------------------------------------------


.
. * Gender
. tab Fetusgender if Laboratoryconfirm==1, freq

      Fetus |
     gender |      Freq.     Percent        Cum.
------------+-----------------------------------
          F |         10       55.56       55.56
          M |          8       44.44      100.00
------------+-----------------------------------
      Total |         18      100.00

. tab Fetusgender if Laboratoryconfirm==0, freq

      Fetus |
     gender |      Freq.     Percent        Cum.
------------+-----------------------------------
          F |         63       56.76       56.76
          M |         48       43.24      100.00
------------+-----------------------------------
      Total |        111      100.00

. gen male=0
```

```
. replace male=1 if Fetusgender=="M"
(58 real changes made)

. tab male Laboratoryconfirm, chi2

           | Laboratory confirmed
           |    Congenital ZIKA
           |       infection
      male |         0          1 |     Total
-----------+----------------------+----------
         0 |        63         10 |        73
         1 |        48          8 |        56
-----------+----------------------+----------
     Total |       111         18 |       129

          Pearson chi2(1) =   0.0091   Pr = 0.924

. stddiff male, by(Laboratoryconfirm)

-----------------------------------------------------------------------------
             |LaboratoryconfirmedCon~0 |LaboratoryconfirmedCon~1 |
             | Mean or N     SD or (%) | Mean or N     SD or (%) |  Std Diff
-------------+-------------------------+-------------------------+------------
        male |     .4324        .49766 |     .4444        .51131 |   -0.02381
-----------------------------------------------------------------------------


.
. * Birth
. sum Term if Laboratoryconfirm==1, d

             Term at birth (weeksâ€™ gestation)
-------------------------------------------------------------
      Percentiles      Smallest
 1%           32             32
 5%           32             37
10%           37             37       Obs                  18
25%           38             37       Sum of Wgt.          18

50%           39                      Mean           38.44444
                        Largest       Std. Dev.       1.94701
75%           39             40
90%           40             40       Variance        3.79085
95%           41             40       Skewness      -2.029757
99%           41             41       Kurtosis        7.79679

. sum Term if Laboratoryconfirm==0, d

             Term at birth (weeksâ€™ gestation)
-------------------------------------------------------------
      Percentiles      Smallest
 1%           36             36
 5%           36             36
10%           37             36       Obs                 111
25%           38             36       Sum of Wgt.         111

50%           39                      Mean           38.73874
                        Largest       Std. Dev.      1.298333
```

```
75%           40             41
90%           40             41       Variance       1.685667
95%           41             41       Skewness      -.2073347
99%           41             41       Kurtosis       2.455612

. ranksum Term, by(Laboratoryconfirm)

Two-sample Wilcoxon rank-sum (Mann-Whitney) test

Laboratory~l |      obs    rank sum    expected
-------------+---------------------------------
           0 |      111        7239        7215
           1 |       18        1146        1170
-------------+---------------------------------
    combined |      129        8385        8385

unadjusted variance    21645.00
adjustment for ties    -1142.03
                     ----------
adjusted variance      20502.97

Ho: Termat~n(Labora~l==0) = Termat~n(Labora~l==1)
             z =   0.168
    Prob > |z| =   0.8669
    Exact Prob =   0.8661

. stddiff Term, by(Laboratoryconfirm)

---------------------------------------------------------------------------
             |LaboratoryconfirmedCon~0 |LaboratoryconfirmedCon~1 |
             | Mean or N     SD or (%) | Mean or N     SD or (%) |  Std Diff
-------------+-------------------------+-------------------------+------------
Termatbirt~n |     38.74        1.2983 |     38.44         1.947 |    0.17785
---------------------------------------------------------------------------


.
. gen prema=0

. replace prema=1 if Term<37
(7 real changes made)

. tab prema Laboratoryconfirm, exact

           | Laboratory confirmed
           |    Congenital ZIKA
           |       infection
     prema |         0          1 |     Total
-----------+----------------------+----------
         0 |       105         17 |       122
         1 |         6          1 |         7
-----------+----------------------+----------
     Total |       111         18 |       129

           Fisher's exact =                 1.000
   1-sided Fisher's exact =                 0.660

. stddiff prema,by(Laboratoryconfirm)
```

```
--------------------------------------------------------------------------
             |LaboratoryconfirmedCon~0 |LaboratoryconfirmedCon~1 |
             | Mean or N    SD or (%) | Mean or N    SD or (%) |  Std Diff
-------------+------------------------+------------------------+------------
       prema |    .05405       .22715 |    .05556        .2357 |   -0.00649
--------------------------------------------------------------------------


.
. tab Mode if Laboratoryconfirm==1, freq

    Mode of |
   delivery |      Freq.     Percent        Cum.
------------+-----------------------------------
         CS |          5       27.78       27.78
          N |         13       72.22      100.00
------------+-----------------------------------
      Total |         18      100.00

. tab Mode if Laboratoryconfirm==0, freq

    Mode of |
   delivery |      Freq.     Percent        Cum.
------------+-----------------------------------
         CS |         14       12.61       12.61
          N |         97       87.39      100.00
------------+-----------------------------------
      Total |        111      100.00

. gen cs=0

. replace cs=1 if Mode=="CS"
(19 real changes made)

. tab cs Laboratoryconfirm, chi2

           | Laboratory confirmed
           |   Congenital ZIKA
           |      infection
        cs |         0          1 |     Total
-----------+----------------------+----------
         0 |        97         13 |       110 
         1 |        14          5 |        19 
-----------+----------------------+----------
     Total |       111         18 |       129 

          Pearson chi2(1) =   2.8362   Pr = 0.092

. stddiff cs, by(Laboratoryconfirm)

--------------------------------------------------------------------------
             |LaboratoryconfirmedCon~0 |LaboratoryconfirmedCon~1 |
             | Mean or N    SD or (%) | Mean or N    SD or (%) |  Std Diff
-------------+------------------------+------------------------+------------
          cs |     .1261        .3335 |     .2778       .46089 |   -0.37699
--------------------------------------------------------------------------


.
```

```
. gen apgar6=0

. replace apgar6=1 if Apgar<6
(15 real changes made)

. tab apgar6 if Laboratoryconfirm==1, freq

     apgar6 |      Freq.     Percent        Cum.
------------+-----------------------------------
          0 |         16       88.89       88.89
          1 |          2       11.11      100.00
------------+-----------------------------------
      Total |         18      100.00

. tab apgar6 if Laboratoryconfirm==0, freq

     apgar6 |      Freq.     Percent        Cum.
------------+-----------------------------------
          0 |         98       88.29       88.29
          1 |         13       11.71      100.00
------------+-----------------------------------
      Total |        111      100.00

. tab apgar6 Laboratoryconfirm, exact

           | Laboratory confirmed
           |    Congenital ZIKA
           |       infection
    apgar6 |         0          1 |     Total
-----------+----------------------+----------
         0 |        98         16 |       114 
         1 |        13          2 |        15 
-----------+----------------------+----------
     Total |       111         18 |       129 

           Fisher's exact =                 1.000
   1-sided Fisher's exact =                 0.651

. stddiff apgar6, by(Laboratoryconfirm)

--------------------------------------------------------------------------
             |LaboratoryconfirmedCon~0 |LaboratoryconfirmedCon~1 |
             | Mean or N     SD or (%) | Mean or N     SD or (%) |  Std Diff
-------------+-------------------------+-------------------------+------------
      apgar6 |     .1171        .32302 |     .1111        .32338 |    0.01858
--------------------------------------------------------------------------


. 
. tab Lactate if Laboratoryconfirm==1, freq

Lactates>4. |
          5 |      Freq.     Percent        Cum.
------------+-----------------------------------
          0 |         16       88.89       88.89
          1 |          2       11.11      100.00
------------+-----------------------------------
      Total |         18      100.00
```

```
. tab Lactate if Laboratoryconfirm==0, freq

Lactates>4. |
          5 |      Freq.     Percent        Cum.
------------+-----------------------------------
          0 |         95       85.59       85.59
          1 |         16       14.41      100.00
------------+-----------------------------------
      Total |        111      100.00

. tab Lactate Laboratoryconfirm, exact

           | Laboratory confirmed
           |    Congenital ZIKA
Lactates>4 |       infection
        .5 |         0          1 |     Total
-----------+----------------------+----------
         0 |        95         16 |       111
         1 |        16          2 |        18
-----------+----------------------+----------
     Total |       111         18 |       129

           Fisher's exact =                 1.000
   1-sided Fisher's exact =                 0.523

. stddiff Lactate, by(Laboratoryconfirm)

--------------------------------------------------------------------------
             |LaboratoryconfirmedCon~0 |LaboratoryconfirmedCon~1 |
             | Mean or N     SD or (%) | Mean or N     SD or (%) |  Std Diff
-------------+-------------------------+-------------------------+------------
  Lactates45 |     .1441        .35283 |     .1111        .32338 |    0.09761
--------------------------------------------------------------------------


.
. log close
      name:  <unnamed>
       log:  \\HOME\lpomar\Mes Documents\Zika_enfants-Guyane\Nature\R1\Dataset\Table1.log
  log type:  text
 closed on:  23 Mar 2021, 11:49:30
------------------------------------------------------------------------------------------------------------------------------------------------------
-----------------------------------
```

```
--------------------------------------------------------------------------------------------------------------------------------------------------------
-----------------------------------
      name:  <unnamed>
       log:  \\HOME\lpomar\Mes Documents\Zika_enfants-Guyane\Nature\R1\Dataset\Table2.log
  log type:  text
 opened on:  23 Mar 2021, 15:02:33

. import excel "\\HOME\lpomar\Mes Documents\Zika_enfants-Guyane\Nature\R1\Dataset\Source Data.xlsx",
sheet("Table 2") firstrow
(26 vars, 132 obs)


.
. destring CongZIKA, replace
CongZIKA: all characters numeric; replaced as byte
(3 missing values generated)

. destring Adverseoutcomesupto2y, replace
Adverseoutcomesupto2y: all characters numeric; replaced as byte
(21 missing values generated)

. destring Neurologicimpairments, replace
Neurologicimpairments: all characters numeric; replaced as byte
(21 missing values generated)

. destring Delayinmotoracquisitions, replace
Delayinmotoracquisitions: all characters numeric; replaced as byte
(21 missing values generated)

. destring Neurosensoryalterations, replace
Neurosensoryalterations: all characters numeric; replaced as byte
(21 missing values generated)

. destring Neurodevelopment2SDat3y, replace
Neurodevelopment2SDat3y: all characters numeric; replaced as byte
(70 missing values generated)

. destring Motordomain2SD, replace
Motordomain2SD: all characters numeric; replaced as byte
(70 missing values generated)

. destring Congitivedomain2SD, replace
Congitivedomain2SD: all characters numeric; replaced as byte
(70 missing values generated)

. destring Socioaffectivedomain2SD, replace
Socioaffectivedomain2SD: all characters numeric; replaced as byte
(70 missing values generated)

. destring Lowsocioeconomicstatus, replace
Lowsocioeconomicstatus: all characters numeric; replaced as byte
(22 missing values generated)


.
. ** Adverse outcomes at 2months of life:
. tab Adverseoutcomesupto2m if CongZIKA==1, freq

    Adverse |
```

```
outcomes up |
     to 2m |      Freq.     Percent        Cum.
------------+-----------------------------------
          0 |         10       55.56       55.56
          1 |          8       44.44      100.00
------------+-----------------------------------
      Total |         18      100.00

. tab Adverseoutcomesupto2m if CongZIKA==0, freq

    Adverse |
outcomes up |
     to 2m |      Freq.     Percent        Cum.
------------+-----------------------------------
          0 |        107       96.40       96.40
          1 |          4        3.60      100.00
------------+-----------------------------------
      Total |        111      100.00

.
.         * Crude Analysis
. glm Adverseoutcomesupto2m CongZIKA, fam(bin) link(log) nolog eform

Generalized linear models                         Number of obs   =        129
Optimization     : ML                             Residual df     =        127
                                                  Scale parameter =          1
Deviance         =  59.17059599                   (1/df) Deviance =   .4659102
Pearson          =          129                   (1/df) Pearson  =   1.015748

Variance function: V(u) = u*(1-u)                 [Bernoulli]
Link function    : g(u) = ln(u)                   [Log]

                                                  AIC             =   .4896945
Log likelihood   = -29.58529799                   BIC             =  -558.0256

------------------------------------------------------------------------------
                      |                 OIM
Adverseoutcomesupto2m | Risk Ratio   Std. Err.      z    P>|z|     [95% Conf. Interval]
----------------------+-------------------------------------------------------
             CongZIKA |   12.33333    6.87173     4.51   0.000      4.13827    36.75718
                _cons |    .036036   .0176904    -6.77   0.000     .0137681    .0943189
------------------------------------------------------------------------------
Note: _cons estimates baseline risk.

. est store CRUDE

.
.         * Adjusted on maternal infection in the 1st trimester
. glm Adverseoutcomesupto2m CongZIKA Maternalinfectioninthe1sttr, fam(bin) link(log) nolog eform

Generalized linear models                         Number of obs   =        129
Optimization     : ML                             Residual df     =        126
                                                  Scale parameter =          1
Deviance         =  49.46063976                   (1/df) Deviance =   .3925448
Pearson          =  139.3390301                   (1/df) Pearson  =   1.105865

Variance function: V(u) = u*(1-u)                 [Bernoulli]
```

```
Link function    : g(u) = ln(u)                  [Log]

                                         AIC             =  .4299274
Log likelihood   = -24.73031988                  BIC             =  -562.8757

------------------------------------------------------------------------------
                             |                 OIM
     Adverseoutcomesupto2m | Risk Ratio   Std. Err.      z    P>|z|     [95% Conf. Interval]
----------------------------+-------------------------------------------------------------
                CongZIKA |   10.07381   5.427441     4.29   0.000      3.50424    28.95966
Maternalinfectioninthe1sttr |   4.170967   2.163393     2.75   0.006     1.509164    11.52755
                   _cons |   .0202444   .0116293    -6.79   0.000     .0065666    .0624127
------------------------------------------------------------------------------
Note: _cons estimates baseline risk.

. est store A

. lrtest CRUDE  /* p=0.0018 => Difference between crude and adjusted RR */

Likelihood-ratio test                              LR chi2(1)  =      9.71
(Assumption: CRUDE nested in A)                        Prob > chi2 =    0.0018

.
.         * Effect modifiers?
. glm Adverseoutcomesupto2m CongZIKA Maternalinfectioninthe1sttr Maternalage, fam(bin) link(log) nolog eform
convergence not achieved

Generalized linear models                          Number of obs   =        129
Optimization     : ML                              Residual df     =        125
                                                   Scale parameter =          1
Deviance         =   49.3541955                    (1/df) Deviance =   .3948336
Pearson          =  125.3919825                    (1/df) Pearson  =   1.003136

Variance function: V(u) = u*(1-u)                  [Bernoulli]
Link function    : g(u) = ln(u)                    [Log]

                                                   AIC             =  .4446062
Log likelihood   = -24.67709775                    BIC             =  -558.1224

------------------------------------------------------------------------------
                             |                 OIM
     Adverseoutcomesupto2m | Risk Ratio   Std. Err.      z    P>|z|     [95% Conf. Interval]
----------------------------+-------------------------------------------------------------
                CongZIKA |   9.294551   4.916121     4.22   0.000     3.296147    26.20899
Maternalinfectioninthe1sttr |   4.250735   2.243583     2.74   0.006     1.510744    11.96017
   Maternalageatbirthyears |   .9689544   .0610639    -0.50   0.617     .8563677    1.096343
                   _cons |   .0481705   .0792893    -1.84   0.065     .0019129    1.213028
------------------------------------------------------------------------------
Note: _cons estimates baseline risk.
Warning: parameter estimates produce inadmissible mean estimates in one or
         more observations.
Warning: convergence not achieved

. lrtest A

Likelihood-ratio test                              LR chi2(1)  =      0.11
(Assumption: A nested in .)                        Prob > chi2 =    0.7442
```

```
. glm Adverseoutcomesupto2m CongZIKA Maternalinfectioninthe1sttr Low, fam(bin) link(log) nolog eform
convergence not achieved

Generalized linear models                 Number of obs   =        107
Optimization     : ML                     Residual df     =        103
                                          Scale parameter =          1
Deviance         =  44.29026976           (1/df) Deviance =   .4300026
Pearson          =  198.6377814           (1/df) Pearson  =   1.928522

Variance function: V(u) = u*(1-u)         [Bernoulli]
Link function    : g(u) = ln(u)           [Log]

                                          AIC             =   .4886941
Log likelihood   = -22.14513488           BIC             =  -437.0111

------------------------------------------------------------------------------------------
                           |                 OIM
     Adverseoutcomesupto2m | Risk Ratio   Std. Err.      z    P>|z|     [95% Conf. Interval]
---------------------------+--------------------------------------------------------------
                  CongZIKA |   12.04362    7.95825     3.77   0.000      3.29834     43.9763
Maternalinfectioninthe1sttr |   5.840566   4.057034     2.54   0.011     1.496872      22.789
    Lowsocioeconomicstatus |   1.553995   1.280183     0.54   0.593     .3091925    7.810347
                     _cons |   .0121336    .011509    -4.65   0.000     .0018906    .0778704
------------------------------------------------------------------------------------------
Note: _cons estimates baseline risk.
Warning: parameter estimates produce inadmissible mean estimates in one or
        more observations.
Warning: convergence not achieved

. lrtest A
observations differ: 107 vs. 129
r(498);

end of do-file

r(498);

. do "C:\Users\lpomar\AppData\Local\Temp\STD3ed0_000000.tmp"

. glm Adverseoutcomesupto2m CongZIKA Maternalinfectioninthe1sttr Alcohol, fam(bin) link(log) nolog eform

Generalized linear models                 Number of obs   =        129
Optimization     : ML                     Residual df     =        125
                                          Scale parameter =          1
Deviance         =  49.34920795           (1/df) Deviance =   .3947937
Pearson          =  143.6365806           (1/df) Pearson  =   1.149093

Variance function: V(u) = u*(1-u)         [Bernoulli]
Link function    : g(u) = ln(u)           [Log]

                                          AIC             =   .4445675
Log likelihood   = -24.67460398           BIC             =  -558.1273

------------------------------------------------------------------------------------------
                           |                 OIM
     Adverseoutcomesupto2m | Risk Ratio   Std. Err.      z    P>|z|     [95% Conf. Interval]
---------------------------+--------------------------------------------------------------
```

```
                CongZIKA |     10.398   5.671159     4.29   0.000     3.57024    30.28325
Maternalinfectioninthe1sttr |   4.311947   2.276365     2.77   0.006     1.532169    12.13501
                 Alcohol |   1.415908   1.389202     0.35   0.723     .2069612    9.686825
                   _cons |   .0189497   .0117243    -6.41   0.000     .0056358    .0637156
------------------------------------------------------------------------------------------
Note: _cons estimates baseline risk.

. lrtest A

Likelihood-ratio test                                 LR chi2(1)  =      0.11
(Assumption: A nested in .)                           Prob > chi2 =    0.7385

.
end of do-file

. do "C:\Users\lpomar\AppData\Local\Temp\STD3ed0_000000.tmp"

. glm Adverseoutcomesupto2m CongZIKA Maternalinfectioninthe1sttr Smoking, fam(bin) link(log) nolog eform

Generalized linear models                         Number of obs   =        128
Optimization     : ML                             Residual df     =        124
                                                  Scale parameter =          1
Deviance         =  48.12350859                   (1/df) Deviance =   .3880928
Pearson          =  161.0006352                   (1/df) Pearson  =   1.298392

Variance function: V(u) = u*(1-u)                 [Bernoulli]
Link function    : g(u) = ln(u)                   [Log]

                                                  AIC             =   .4384649
Log likelihood   = -24.06175429                   BIC             =  -553.5282

------------------------------------------------------------------------------------------
                            |                 OIM
      Adverseoutcomesupto2m | Risk Ratio   Std. Err.      z    P>|z|     [95% Conf. Interval]
----------------------------+-------------------------------------------------------------
                CongZIKA |   12.59692   7.730123     4.13   0.000     3.783756    41.93781
Maternalinfectioninthe1sttr |   3.982913   2.037706     2.70   0.007     1.461221     10.8564
                 Smoking |   4.146219   4.336732     1.36   0.174     .5337515    32.20812
                   _cons |    .016896   .0107137    -6.44   0.000     .0048757    .0585501
------------------------------------------------------------------------------------------
Note: _cons estimates baseline risk.

. lrtest A
observations differ: 128 vs. 129
r(498);

end of do-file

r(498);

. do "C:\Users\lpomar\AppData\Local\Temp\STD3ed0_000000.tmp"

. glm Adverseoutcomesupto2m CongZIKA Maternalinfectioninthe1sttr Leadp, fam(bin) link(log) nolog eform
note: Leadpoisoning != 0 predicts failure perfectly
      Leadpoisoning dropped and 7 obs not used
```

```
Generalized linear models                          Number of obs   =        122
Optimization     : ML                          Residual df     =        126
                                               Scale parameter =          1
Deviance         =  49.49622339                    (1/df) Deviance =   .3928272
Pearson          =  131.2625822                    (1/df) Pearson  =   1.041767

Variance function: V(u) = u*(1-u)                  [Bernoulli]
Link function    : g(u) = ln(u)                    [Log]

                                               AIC             =     .42423
Log likelihood   = -24.36283646                    BIC             =  -562.8401

------------------------------------------------------------------------------
                         |                 OIM
     Adverseoutcomesupto2m | Risk Ratio   Std. Err.      z    P>|z|     [95% Conf. Interval]
---------------------------+--------------------------------------------------
                CongZIKA |   10.13224   5.458601     4.30   0.000     3.524788    29.12583
Maternalinfectioninthe1sttr |   3.820946   1.968834     2.60   0.009     1.391775    10.48994
            Leadpoisoning |          1  (omitted)
                   _cons |   .0219793   .0125777    -6.67   0.000       .00716    .0674706
------------------------------------------------------------------------------
Note: _cons estimates baseline risk.

. lrtest A
df(unrestricted) = df(restricted) = 3
r(498);

end of do-file

r(498);

. do "C:\Users\lpomar\AppData\Local\Temp\STD3ed0_000000.tmp"

. glm Adverseoutcomesupto2m CongZIKA Maternalinfectioninthe1sttr Anymaternalco, fam(bin) link(log) nolog
eform
note: Anymaternalcomorbidities != 0 predicts failure perfectly
      Anymaternalcomorbidities dropped and 18 obs not used


Generalized linear models                          Number of obs   =        111
Optimization     : ML                          Residual df     =        126
                                               Scale parameter =          1
Deviance         =  49.77844925                    (1/df) Deviance =   .3950671
Pearson          =  114.0857765                    (1/df) Pearson  =   .9054427

Variance function: V(u) = u*(1-u)                  [Bernoulli]
Link function    : g(u) = ln(u)                    [Log]

                                               AIC             =   .4082759
Log likelihood   =  -23.3337971                    BIC             =  -562.5579

------------------------------------------------------------------------------
                         |                 OIM
     Adverseoutcomesupto2m | Risk Ratio   Std. Err.      z    P>|z|     [95% Conf. Interval]
---------------------------+--------------------------------------------------
                CongZIKA |   9.591073   5.279054     4.11   0.000     3.261029    28.20849
Maternalinfectioninthe1sttr |   3.351702   1.754452     2.31   0.021     1.201443    9.350347
```

```
   Anymaterna lcomorbidities |          1  (omitted)
                  _cons |   .0266267     .01488    -6.49   0.000      .008905    .0796159
------------------------------------------------------------------------------------------
Note: _cons estimates baseline risk.

. lrtest A
df(unrestricted) = df(restricted) = 3
r(498);

end of do-file

r(498);

. do "C:\Users\lpomar\AppData\Local\Temp\STD3ed0_000000.tmp"

. glm Adverseoutcomesupto2m CongZIKA Maternalinfectioninthe1sttr Infantgender, fam(bin) link(log) nolog eform
convergence not achieved

Generalized linear models                         Number of obs   =        129
Optimization     : ML                             Residual df     =        125
                                                  Scale parameter =          1
Deviance         =  50.13978181                   (1/df) Deviance =   .4011183
Pearson          =  213.6708675                   (1/df) Pearson  =   1.709367

Variance function: V(u) = u*(1-u)                 [Bernoulli]
Link function    : g(u) = ln(u)                   [Log]

                                                  AIC             =    .450696
Log likelihood   = -25.06989091                   BIC             =  -557.3368

------------------------------------------------------------------------------------------
                           |                 OIM
     Adverseoutcomesupto2m | Risk Ratio   Std. Err.      z    P>|z|     [95% Conf. Interval]
---------------------------+--------------------------------------------------------------
                  CongZIKA |   16.11583   11.18632     4.00   0.000     4.134438    62.81866
Maternalinfectioninthe1sttr |   5.927055   4.261905     2.47   0.013     1.448019    24.26071
              Infantgender |    1.79128   1.395039     0.75   0.454     .3892715    8.242795
                     _cons |   .0090272   .0100989    -4.21   0.000     .0010076    .0808742
------------------------------------------------------------------------------------------
Note: _cons estimates baseline risk.
Warning: parameter estimates produce inadmissible mean estimates in one or
         more observations.
Warning: convergence not achieved

. lrtest A

Likelihood-ratio test                             LR chi2(1)  =     -0.68
(Assumption: A nested in .)                       Prob > chi2 =    1.0000

.
end of do-file

. do "C:\Users\lpomar\AppData\Local\Temp\STD3ed0_000000.tmp"

. glm Adverseoutcomesupto2m CongZIKA Maternalinfectioninthe1sttr Twins, fam(bin) link(log) nolog eform
note: Twins != 0 predicts failure perfectly
      Twins dropped and 8 obs not used
```

```
Generalized linear models                     Number of obs   =        121
Optimization     : ML                         Residual df     =        126
                                              Scale parameter =          1
Deviance         =  49.57789331               (1/df) Deviance =   .3934753
Pearson          =  127.8620699               (1/df) Pearson  =   1.014778

Variance function: V(u) = u*(1-u)             [Bernoulli]
Link function    : g(u) = ln(u)               [Log]

                                              AIC             =   .4201519
Log likelihood   = -24.09979754               BIC             =  -562.7585

------------------------------------------------------------------------------------------
                          |                 OIM
    Adverseoutcomesupto2m | Risk Ratio   Std. Err.      z    P>|z|     [95% Conf. Interval]
--------------------------+---------------------------------------------------------------
                 CongZIKA |   10.47541   5.685295     4.33   0.000     3.615764    30.34881
Maternalinfectioninthe1sttr |   3.546996   1.830741     2.45   0.014     1.289801    9.754357
                    Twins |          1  (omitted)
                    _cons |   .0229467   .0130249    -6.65   0.000     .0075434    .0698033
------------------------------------------------------------------------------------------
Note: _cons estimates baseline risk.

. lrtest A
df(unrestricted) = df(restricted) = 3
r(498);

end of do-file

r(498);

. do "C:\Users\lpomar\AppData\Local\Temp\STD3ed0_000000.tmp"

. glm Adverseoutcomesupto2m CongZIKA Maternalinfectioninthe1sttr Modeofdelivery, fam(bin) link(log) nolog
eform
convergence not achieved

Generalized linear models                     Number of obs   =        129
Optimization     : ML                         Residual df     =        127
                                              Scale parameter =          1
Deviance         =  104.6869905               (1/df) Deviance =    .824307
Pearson          =  100000049.2               (1/df) Pearson  =     787402

Variance function: V(u) = u*(1-u)             [Bernoulli]
Link function    : g(u) = ln(u)               [Log]

                                              AIC             =   .8425348
Log likelihood   = -52.34349525               BIC             =  -512.5092

------------------------------------------------------------------------------------------
                          |                 OIM
    Adverseoutcomesupto2m | Risk Ratio   Std. Err.      z    P>|z|     [95% Conf. Interval]
--------------------------+---------------------------------------------------------------
                 CongZIKA |   3.810155          .        .       .            .           .
Maternalinfectioninthe1sttr |   2.028606          .        .       .            .           .
           Modeofdelivery |   .3800644   .2491885    -1.48   0.140     .1051401    1.373871
```

```
                    _cons |   .1437552   1.44e-09 -1.9e+08   0.000     .1437552    .1437552
------------------------------------------------------------------------------------------
Note: _cons estimates baseline risk.
Warning: parameter estimates produce inadmissible mean estimates in one or
         more observations.
Warning: convergence not achieved

. lrtest A

Likelihood-ratio test                                 LR chi2(1)  =     55.23
(Assumption: . nested in A)                           Prob > chi2 =    0.0000

.
end of do-file

. do "C:\Users\lpomar\AppData\Local\Temp\STD3ed0_000000.tmp"

. glm Adverseoutcomesupto2m CongZIKA Maternalinfectioninthe1sttr Prematurity, fam(bin) link(log) nolog eform
convergence not achieved

Generalized linear models                          Number of obs   =        129
Optimization     : ML                              Residual df     =        125
                                                   Scale parameter =          1
Deviance         =  51.14091788                    (1/df) Deviance =   .4091273
Pearson          =   185.997202                    (1/df) Pearson  =   1.487978

Variance function: V(u) = u*(1-u)                  [Bernoulli]
Link function    : g(u) = ln(u)                    [Log]

                                                   AIC             =   .4584567
Log likelihood   = -25.57045894                    BIC             =  -556.3356

------------------------------------------------------------------------------------------
                            |                 OIM
      Adverseoutcomesupto2m | Risk Ratio   Std. Err.      z    P>|z|     [95% Conf. Interval]
----------------------------+-------------------------------------------------------------
                   CongZIKA |   11.95889   7.362507     4.03   0.000     3.578058    39.97001
Maternalinfectioninthe1sttr |   4.878225   2.911104     2.66   0.008     1.514619     15.7116
           Prematurity37wg |   9.984096   11.48523     2.00   0.045     1.047435    95.16785
                      _cons |   .0143084   .0097692    -6.22   0.000     .0037534    .0545454
------------------------------------------------------------------------------------------
Note: _cons estimates baseline risk.
Warning: parameter estimates produce inadmissible mean estimates in one or
         more observations.
Warning: convergence not achieved

. lrtest A

Likelihood-ratio test                                 LR chi2(1)  =     -1.68
(Assumption: A nested in .)                           Prob > chi2 =    1.0000

.
end of do-file

. do "C:\Users\lpomar\AppData\Local\Temp\STD3ed0_000000.tmp"

. tab Neontaldemise CongZIKA, exact
```

```
   Neontal |       CongZIKA
    demise |         0          1 |     Total
-----------+----------------------+----------
         0 |       111         17 |       128 
         1 |         0          1 |         1 
-----------+----------------------+----------
     Total |       111         18 |       129 

           Fisher's exact =                 0.140
   1-sided Fisher's exact =                 0.140

. 
. glm Structuralbrain CongZIKA, fam(bin) link(log) nolog eform

Generalized linear models                         Number of obs   =        129
Optimization     : ML                             Residual df     =        127
                                                  Scale parameter =          1
Deviance         =  50.49819596                   (1/df) Deviance =   .3976236
Pearson          =          129                   (1/df) Pearson  =   1.015748

Variance function: V(u) = u*(1-u)                 [Bernoulli]
Link function    : g(u) = ln(u)                   [Log]

                                                  AIC             =   .4224666
Log likelihood   = -25.24909798                   BIC             =   -566.698

-------------------------------------------------------------------------------------------
                         |                 OIM
Structuralbrainanomalies | Risk Ratio   Std. Err.      z    P>|z|     [95% Conf. Interval]
-------------------------+-----------------------------------------------------------------
                CongZIKA |   12.33333   8.138462     3.81   0.000     3.383713     44.9539
                   _cons |    .027027   .0153918    -6.34   0.000      .008852    .0825188
-------------------------------------------------------------------------------------------
Note: _cons estimates baseline risk.

. glm Structuralbrain CongZIKA Maternalinfectioninthe1sttr, fam(bin) link(log) nolog eform

Generalized linear models                         Number of obs   =        129
Optimization     : ML                             Residual df     =        126
                                                  Scale parameter =          1
Deviance         =  43.76757084                   (1/df) Deviance =   .3473617
Pearson          =   167.458276                   (1/df) Pearson  =   1.329034

Variance function: V(u) = u*(1-u)                 [Bernoulli]
Link function    : g(u) = ln(u)                   [Log]

                                                  AIC             =   .3857951
Log likelihood   = -21.88378542                   BIC             =  -568.5688

----------------------------------------------------------------------------------------------
                            |                 OIM
   Structuralbrainanomalies | Risk Ratio   Std. Err.      z    P>|z|     [95% Conf. Interval]
----------------------------+-----------------------------------------------------------------
                   CongZIKA |   10.49826    6.71045     3.68   0.000     2.999385    36.74534
Maternalinfectioninthe1sttr |   4.454848   2.779989     2.39   0.017     1.311148     15.1361
                      _cons |    .014491   .0098654    -6.22   0.000      .003816     .055029
----------------------------------------------------------------------------------------------
```

```
Note: _cons estimates baseline risk.

.
. glm Neurologicalsymptoms CongZIKA, fam(bin) link(log) nolog eform

Generalized linear models                     Number of obs   =        129
Optimization     : ML                         Residual df     =        127
                                              Scale parameter =          1
Deviance         =  32.68034508               (1/df) Deviance =   .2573256
Pearson          =  128.9999999               (1/df) Pearson  =   1.015748

Variance function: V(u) = u*(1-u)             [Bernoulli]
Link function    : g(u) = ln(u)               [Log]

                                              AIC             =   .2843438
Log likelihood   = -16.34017254               BIC             =  -584.5158

------------------------------------------------------------------------------
                     |                 OIM
Neurologicalsymptoms | Risk Ratio   Std. Err.      z    P>|z|     [95% Conf. Interval]
---------------------+--------------------------------------------------------
            CongZIKA |   30.83333   32.85502     3.22   0.001     3.819506    248.9051
               _cons |    .009009   .0089683    -4.73   0.000     .0012803    .0633922
------------------------------------------------------------------------------
Note: _cons estimates baseline risk.

. glm Neurologicalsymptoms CongZIKA Maternalinfectioninthe1sttr, fam(bin) link(log) nolog eform

Generalized linear models                     Number of obs   =        129
Optimization     : ML                         Residual df     =        126
                                              Scale parameter =          1
Deviance         =   25.5258302               (1/df) Deviance =    .202586
Pearson          =  57.99262822               (1/df) Pearson  =    .460259

Variance function: V(u) = u*(1-u)             [Bernoulli]
Link function    : g(u) = ln(u)               [Log]

                                              AIC             =   .2443863
Log likelihood   =  -12.7629151               BIC             =  -586.8105

---------------------------------------------------------------------------------------------
                            |                 OIM
       Neurologicalsymptoms | Risk Ratio   Std. Err.      z    P>|z|     [95% Conf. Interval]
----------------------------+----------------------------------------------------------------
                   CongZIKA |    21.4455   22.58241     2.91   0.004     2.722759     168.913
Maternalinfectioninthe1sttr |   8.609643   8.936448     2.07   0.038     1.125832    65.84103
                      _cons |   .0031805   .0039773    -4.60   0.000     .0002742     .036893
---------------------------------------------------------------------------------------------
Note: _cons estimates baseline risk.


.
.                       ** Structural brain anomalies as effect-modifier?
. glm Neurologicalsymptoms CongZIKA Maternalinfectioninthe1sttr Structuralbrain, fam(bin) link(log) nolog eform

Generalized linear models                     Number of obs   =        129
Optimization     : ML                         Residual df     =        125
                                              Scale parameter =          1
```

```
Deviance         =  25.52492504                 (1/df) Deviance =   .2041994
Pearson          =  58.15185761                 (1/df) Pearson  =   .4652149

Variance function: V(u) = u*(1-u)               [Bernoulli]
Link function    : g(u) = ln(u)                 [Log]

                                                AIC             =   .2598831
Log likelihood   = -12.76246252                 BIC             =  -581.9516

------------------------------------------------------------------------------------------
                          |                 OIM
      Neurologicalsymptoms | Risk Ratio   Std. Err.      z    P>|z|     [95% Conf. Interval]
---------------------------+--------------------------------------------------------------
                CongZIKA |   21.23418   23.46672     2.76   0.006     2.434106    185.2386
Maternalinfectioninthe1sttr |   8.534267   9.216956     1.99   0.047     1.027717    70.86939
  Structuralbrainanomalies |   1.019548   .6584403     0.03   0.976      .287533    3.615157
                   _cons |   .0031989   .0040463    -4.54   0.000     .0002681     .038166
------------------------------------------------------------------------------------------
Note: _cons estimates baseline risk.

. tab Neurologicalsymptoms CongZIKA if Structuralbrain==1, exact

Neurologic |
        al |       CongZIKA
  symptoms |         0          1 |     Total
-----------+----------------------+----------
         0 |         3          3 |         6
         1 |         0          3 |         3
-----------+----------------------+----------
     Total |         3          6 |         9

           Fisher's exact =                 0.464
   1-sided Fisher's exact =                 0.238

. glm Neurologicalsymptoms CongZIKA if Structuralbrain==0, fam(bin) link(log) nolog eform

Generalized linear models                         Number of obs   =        120
Optimization     : ML                             Residual df     =        118
                                                  Scale parameter =          1
Deviance         =   22.1684435                   (1/df) Deviance =   .1878682
Pearson          =          120                   (1/df) Pearson  =   1.016949

Variance function: V(u) = u*(1-u)                 [Bernoulli]
Link function    : g(u) = ln(u)                   [Log]

                                                  AIC             =   .2180704
Log likelihood   = -11.08422175                   BIC             =  -542.7556

--------------------------------------------------------------------------------------
                     |                 OIM
Neurologicalsymptoms | Risk Ratio   Std. Err.      z    P>|z|     [95% Conf. Interval]
---------------------+----------------------------------------------------------------
            CongZIKA |         18   21.35416     2.44   0.015     1.759772    184.1148
               _cons |   .0092593   .0092163    -4.70   0.000     .0013162     .065137
--------------------------------------------------------------------------------------
Note: _cons estimates baseline risk.
```

```
. glm Neurologicalsymptoms CongZIKA Maternalinfectioninthe1sttr if Structuralbrain==0, fam(bin) link(log) nolog
eform

Generalized linear models                         Number of obs   =        120
Optimization     : ML                             Residual df     =        117
                                                  Scale parameter =          1
Deviance         =  18.57082858                   (1/df) Deviance =    .158725
Pearson          =  51.83984929                   (1/df) Pearson  =   .4430756

Variance function: V(u) = u*(1-u)                 [Bernoulli]
Link function    : g(u) = ln(u)                   [Log]

                                                  AIC             =   .2047569
Log likelihood   =  -9.28541429                   BIC             =  -541.5657

----------------------------------------------------------------------------------------------
                             |                 OIM
        Neurologicalsymptoms | Risk Ratio   Std. Err.      z    P>|z|     [95% Conf. Interval]
-----------------------------+----------------------------------------------------------------
                    CongZIKA |   20.49089   22.96574     2.69   0.007     2.277994    184.3185
Maternalinfectioninthe1sttr |   7.737567   8.565827     1.85   0.065     .8836632    67.75199
                       _cons |   .0035561      .0045    -4.46   0.000     .0002977    .0424727
----------------------------------------------------------------------------------------------
Note: _cons estimates baseline risk.

. 
end of do-file

. do "C:\Users\lpomar\AppData\Local\Temp\STD3ed0_000000.tmp"

. 
.                         ** Stratified on Maternal infection in the 1st trimester
. cs Adverseoutcomesupto2m CongZIKA, by(Maternalinfectioninthe1sttr)

Maternal infecti |       RR       [95% Conf. Interval]   M-H Weight
-----------------+-------------------------------------------------
               0 |    7.636364     1.192969   48.88143     .2315789 
               1 |    11.57143     2.947891   45.42161     .4117647 
-----------------+-------------------------------------------------
           Crude |    12.33333      4.13827   36.75718
    M-H combined |    10.15496     3.382333   30.48876
-------------------------------------------------------------------
Test of homogeneity (M-H)      chi2(1) =    0.126  Pr>chi2 = 0.7231


. 
end of do-file

. do "C:\Users\lpomar\AppData\Local\Temp\STD3ed0_000000.tmp"


. 
. ** Adverse outcomes at 2years of life:
. glm Adverseoutcomesupto2y CongZIKA, fam(bin) link(log) nolog eform

Generalized linear models                         Number of obs   =        111
Optimization     : ML                             Residual df     =        109
                                                  Scale parameter =          1
Deviance         =  59.47439346                   (1/df) Deviance =   .5456366
```

```
Pearson          =         111                  (1/df) Pearson  =   1.018349

Variance function: V(u) = u*(1-u/1)              [Binomial]
Link function    : g(u) = ln(u)                  [Log]

                                 AIC            =  .5718414
Log likelihood   = -29.73719673                  BIC            =  -453.8644

------------------------------------------------------------------------------
                   |                 OIM
Adverseoutcomesupto2y | Risk Ratio   Std. Err.      z    P>|z|     [95% Conf. Interval]
-------------------+----------------------------------------------------------
           CongZIKA |       7.68   4.132834     3.79   0.000     2.674882    22.05047
              _cons |   .0520833   .0226777    -6.79   0.000     .0221858    .1222706
------------------------------------------------------------------------------
Note: _cons estimates baseline risk.

. est store CRUDE

.
.         * Adjusted on maternal infection in the 1st trimester
. glm Adverseoutcomesupto2y CongZIKA Maternalinfectioninthe1sttr, fam(bin) link(log) nolog eform

Generalized linear models                          Number of obs   =        111
Optimization     : ML                              Residual df     =        108
                                                   Scale parameter =          1
Deviance         =  58.28203098                    (1/df) Deviance =   .5396484
Pearson          =  107.3983993                    (1/df) Pearson  =   .9944296

Variance function: V(u) = u*(1-u/1)              [Binomial]
Link function    : g(u) = ln(u)                  [Log]

                                 AIC            =  .5791174
Log likelihood   = -29.14101549                  BIC            =  -450.3472

------------------------------------------------------------------------------------
                         |                 OIM
     Adverseoutcomesupto2y | Risk Ratio   Std. Err.      z    P>|z|     [95% Conf. Interval]
-------------------------+----------------------------------------------------------
                 CongZIKA |   6.667957   3.729054     3.39   0.001     2.228222    19.95387
Maternalinfectioninthe1sttr |    1.78164   .9387635     1.10   0.273     .6343274    5.004104
                    _cons |   .0449262   .0209486    -6.65   0.000     .0180133    .1120485
------------------------------------------------------------------------------------
Note: _cons estimates baseline risk.

. est store A

. lrtest CRUDE

Likelihood-ratio test                             LR chi2(1)  =      1.19
(Assumption: CRUDE nested in A)                   Prob > chi2 =    0.2749

.
.         * Effect modifiers?
. glm Adverseoutcomesupto2y CongZIKA Maternalinfectioninthe1sttr Maternalage, fam(bin) link(log) nolog eform

Generalized linear models                          Number of obs   =        111
```

```
Optimization     : ML                          Residual df     =        107
                                            Scale parameter =          1
Deviance         =  58.28068961                (1/df) Deviance =   .5446793
Pearson          =   107.819364                (1/df) Pearson  =   1.007658

Variance function: V(u) = u*(1-u/1)            [Binomial]
Link function    : g(u) = ln(u)                [Log]

                                            AIC             =   .5971233
Log likelihood   = -29.14034481                BIC             =   -445.639

------------------------------------------------------------------------------
                           |                 OIM
     Adverseoutcomesupto2y | Risk Ratio   Std. Err.      z    P>|z|     [95% Conf. Interval]
---------------------------+--------------------------------------------------------------
                 CongZIKA |   6.682789   3.758764     3.38   0.001     2.219196    20.12425
Maternalinfectioninthe1sttr |   1.785488   .9472611     1.09   0.275     .6311988    5.050656
   Maternalageatbirthyears |   .9982092   .0489371    -0.04   0.971     .9067583    1.098883
                     _cons |   .0470907   .0643476    -2.24   0.025     .0032345    .6855956
------------------------------------------------------------------------------
Note: _cons estimates baseline risk.

. lrtest A

Likelihood-ratio test                          LR chi2(1)  =      0.00
(Assumption: A nested in .)                    Prob > chi2 =    0.9708

. glm Adverseoutcomesupto2y CongZIKA Maternalinfectioninthe1sttr Low, fam(bin) link(log) nolog eform

Generalized linear models                      Number of obs   =         93
Optimization     : ML                          Residual df     =         89
                                            Scale parameter =          1
Deviance         =   53.6510958                (1/df) Deviance =   .6028213
Pearson          =  87.58781243                (1/df) Pearson  =   .9841327

Variance function: V(u) = u*(1-u/1)            [Binomial]
Link function    : g(u) = ln(u)                [Log]

                                            AIC             =    .662915
Log likelihood   =  -26.8255479                BIC             =  -349.7503

------------------------------------------------------------------------------
                           |                 OIM
     Adverseoutcomesupto2y | Risk Ratio   Std. Err.      z    P>|z|     [95% Conf. Interval]
---------------------------+--------------------------------------------------------------
                 CongZIKA |   6.628856   3.712668     3.38   0.001     2.211568    19.86904
Maternalinfectioninthe1sttr |    1.36452    .696087     0.61   0.542     .5020575    3.708567
    Lowsocioeconomicstatus |   1.716076   .8379849     1.11   0.269     .6589952    4.468797
                     _cons |   .0449557   .0232193    -6.01   0.000     .0163359    .1237158
------------------------------------------------------------------------------
Note: _cons estimates baseline risk.

. lrtest A
observations differ: 93 vs. 111
r(498);

end of do-file
```

```
r(498);

. do "C:\Users\lpomar\AppData\Local\Temp\STD3ed0_000000.tmp"

. glm Adverseoutcomesupto2y CongZIKA Maternalinfectioninthe1sttr Alcohol, fam(bin) link(log) nolog eform

Generalized linear models                          Number of obs   =        111
Optimization     : ML                              Residual df     =        107
                                                   Scale parameter =          1
Deviance         =  58.2782245                     (1/df) Deviance =   .5446563
Pearson          =  107.3874291                    (1/df) Pearson  =   1.003621

Variance function: V(u) = u*(1-u/1)                [Binomial]
Link function    : g(u) = ln(u)                    [Log]

                                                   AIC             =   .5971011
Log likelihood   = -29.13911225                    BIC             =  -445.6415

------------------------------------------------------------------------------------------
                            |                 OIM
      Adverseoutcomesupto2y | Risk Ratio   Std. Err.      z    P>|z|     [95% Conf. Interval]
----------------------------+-------------------------------------------------------------
                   CongZIKA |   6.652203   3.733947     3.38   0.001     2.214001    19.98726
Maternalinfectioninthe1sttr |   1.772124   .9465195     1.07   0.284     .6220911    5.048174
                    Alcohol |   .9425184   .9145275    -0.06   0.951     .1407237     6.31266
                      _cons |   .0453355   .0221073    -6.34   0.000     .0174325    .1179009
------------------------------------------------------------------------------------------
Note: _cons estimates baseline risk.

. lrtest A

Likelihood-ratio test                              LR chi2(1)  =      0.00
(Assumption: A nested in .)                        Prob > chi2 =    0.9508

. glm Adverseoutcomesupto2y CongZIKA Maternalinfectioninthe1sttr Smoking, fam(bin) link(log) nolog eform
note: Smoking != 0 predicts failure perfectly
      Smoking dropped and 4 obs not used


Generalized linear models                          Number of obs   =        106
Optimization     : ML                              Residual df     =        107
                                                   Scale parameter =          1
Deviance         =  58.23854677                    (1/df) Deviance =   .5442855
Pearson          =  102.9291785                    (1/df) Pearson  =   .9619549

Variance function: V(u) = u*(1-u/1)                [Binomial]
Link function    : g(u) = ln(u)                    [Log]

                                                   AIC             =    .573422
Log likelihood   = -28.53821188                    BIC             =  -444.7129

------------------------------------------------------------------------------------------
                            |                 OIM
      Adverseoutcomesupto2y | Risk Ratio   Std. Err.      z    P>|z|     [95% Conf. Interval]
----------------------------+-------------------------------------------------------------
                   CongZIKA |   6.847706   3.851057     3.42   0.001     2.274264    20.61813
Maternalinfectioninthe1sttr |   1.641937   .8656024     0.94   0.347     .5842746    4.614196
```

```
                  Smoking |          1  (omitted)
                    _cons |   .0481465   .0222375    -6.57   0.000     .0194724    .1190447
------------------------------------------------------------------------------------------
Note: _cons estimates baseline risk.

. lrtest A
df(unrestricted) = df(restricted) = 3
r(498);

end of do-file

r(498);

. do "C:\Users\lpomar\AppData\Local\Temp\STD3ed0_000000.tmp"

. glm Adverseoutcomesupto2y CongZIKA Maternalinfectioninthe1sttr Leadp, fam(bin) link(log) nolog eform
convergence not achieved

Generalized linear models                         Number of obs   =        111
Optimization     : ML                             Residual df     =        109
                                                  Scale parameter =          1
Deviance         =  108.2702414                   (1/df) Deviance =    .993305
Pearson          =  100000057.9                   (1/df) Pearson  =   917431.7

Variance function: V(u) = u*(1-u/1)               [Binomial]
Link function    : g(u) = ln(u)                   [Log]

                                                  AIC             =   1.011444
Log likelihood   = -54.13512068                   BIC             =  -405.0686

------------------------------------------------------------------------------------------
                            |                 OIM
      Adverseoutcomesupto2y | Risk Ratio   Std. Err.      z    P>|z|     [95% Conf. Interval]
----------------------------+-------------------------------------------------------------
                   CongZIKA |    3.46651          .        .       .            .           .
Maternalinfectioninthe1sttr |   .7715198   .2863655    -0.70   0.485       .37274    1.596938
              Leadpoisoning |   2.432884          .        .       .            .           .
                      _cons |    .165099   1.65e-09 -1.8e+08   0.000      .165099     .165099
------------------------------------------------------------------------------------------
Note: _cons estimates baseline risk.
Warning: parameter estimates produce inadmissible mean estimates in one or
         more observations.
Warning: convergence not achieved


.
end of do-file

. Tab Adverseoutcomesupto2y CongZIKA if Leadp==1
command Tab is unrecognized
r(199);

. tab Adverseoutcomesupto2y CongZIKA if Leadp==1

   Adverse |
  outcomes |       CongZIKA
  up to 2y |         0          1 |     Total
-----------+----------------------+----------
```

```
        0 |         4          1 |         5 
        1 |         1          0 |         1 
-----------+----------------------+----------
     Total |         5          1 |         6 

. tab Adverseoutcomesupto2y CongZIKA if Leadp==0

   Adverse |
  outcomes |       CongZIKA
  up to 2y |         0          1 |     Total
-----------+----------------------+----------
         0 |        87          8 |        95 
         1 |         4          6 |        10 
-----------+----------------------+----------
     Total |        91         14 |       105 

. do "C:\Users\lpomar\AppData\Local\Temp\STD3ed0_000000.tmp"

. glm Adverseoutcomesupto2y CongZIKA Maternalinfectioninthe1sttr Anymaternalco, fam(bin) link(log) nolog
eform
note: Anymaternalcomorbidities != 0 predicts failure perfectly
      Anymaternalcomorbidities dropped and 16 obs not used


Generalized linear models                          Number of obs   =         95
Optimization     : ML                              Residual df     =        108
                                                   Scale parameter =          1
Deviance         =  59.03125837                    (1/df) Deviance =   .5465857
Pearson          =  94.72467703                    (1/df) Pearson  =   .8770803

Variance function: V(u) = u*(1-u/1)                [Binomial]
Link function    : g(u) = ln(u)                    [Log]

                                                   AIC             =   .5414375
Log likelihood   =  -27.0497829                    BIC             =   -449.598

---------------------------------------------------------------------------------------------
                            |                 OIM
      Adverseoutcomesupto2y | Risk Ratio   Std. Err.      z    P>|z|     [95% Conf. Interval]
----------------------------+----------------------------------------------------------------
                   CongZIKA |   7.374224   4.233645     3.48   0.001     2.393479    22.71972
Maternalinfectioninthe1sttr |   1.343991   .7123262     0.56   0.577     .4756121     3.79787
   Anymaternalcomorbidities |          1  (omitted)
                      _cons |    .056681   .0255512    -6.37   0.000     .0234274    .1371358
---------------------------------------------------------------------------------------------
Note: _cons estimates baseline risk.

. lrtest A
df(unrestricted) = df(restricted) = 3
r(498);

end of do-file

r(498);

. do "C:\Users\lpomar\AppData\Local\Temp\STD3ed0_000000.tmp"
```

```
. glm Adverseoutcomesupto2y CongZIKA Maternalinfectioninthe1sttr Infantgender, fam(bin) link(log) nolog eform

Generalized linear models                  Number of obs   =        111
Optimization     : ML                      Residual df     =        107
                                           Scale parameter =          1
Deviance         =  57.75156168            (1/df) Deviance =   .5397342
Pearson          =  108.0139714            (1/df) Pearson  =   1.009476

Variance function: V(u) = u*(1-u/1)        [Binomial]
Link function    : g(u) = ln(u)            [Log]

                                           AIC             =   .5923564
Log likelihood   = -28.87578084            BIC             =  -446.1682

---------------------------------------------------------------------------------------------
                            |                 OIM
      Adverseoutcomesupto2y | Risk Ratio   Std. Err.      z    P>|z|     [95% Conf. Interval]
----------------------------+----------------------------------------------------------------
                   CongZIKA |   6.499541    3.62741     3.35   0.001     2.176832     19.4062
Maternalinfectioninthe1sttr |   1.836386   .9526067     1.17   0.241     .6643724    5.075937
               Infantgender |   .6968215   .3475328    -0.72   0.469     .2621762    1.852038
                      _cons |   .0538296   .0280132    -5.61   0.000     .0194111    .1492765
---------------------------------------------------------------------------------------------
Note: _cons estimates baseline risk.

. lrtest A

Likelihood-ratio test                                 LR chi2(1)  =      0.53
(Assumption: A nested in .)                           Prob > chi2 =    0.4664

.
end of do-file

. do "C:\Users\lpomar\AppData\Local\Temp\STD3ed0_000000.tmp"

. glm Adverseoutcomesupto2y CongZIKA Maternalinfectioninthe1sttr Twins, fam(bin) link(log) nolog eform
note: Twins != 0 predicts failure perfectly
      Twins dropped and 5 obs not used


Generalized linear models                  Number of obs   =        106
Optimization     : ML                      Residual df     =        108
                                           Scale parameter =          1
Deviance         =  58.29501464            (1/df) Deviance =   .5397687
Pearson          =  103.3580007            (1/df) Pearson  =   .9570185

Variance function: V(u) = u*(1-u/1)        [Binomial]
Link function    : g(u) = ln(u)            [Log]

                                           AIC             =   .5748637
Log likelihood   = -28.90493481            BIC             =  -450.3342

---------------------------------------------------------------------------------------------
                            |                 OIM
      Adverseoutcomesupto2y | Risk Ratio   Std. Err.      z    P>|z|     [95% Conf. Interval]
----------------------------+----------------------------------------------------------------
                   CongZIKA |   6.417676    3.56332     3.35   0.001     2.161527    19.05438
```

```
Maternalinfectioninthe1sttr |   1.743244    .912558     1.06   0.288    .6248401    4.863483
                      Twins |          1  (omitted)
                      _cons |   .0473565   .0221332    -6.53   0.000    .0189474    .1183609
------------------------------------------------------------------------------------------
Note: _cons estimates baseline risk.

. lrtest A
df(unrestricted) = df(restricted) = 3
r(498);

end of do-file

r(498);

. do "C:\Users\lpomar\AppData\Local\Temp\STD3ed0_000000.tmp"

. glm Adverseoutcomesupto2y CongZIKA Maternalinfectioninthe1sttr Modeofdelivery, fam(bin) link(log) nolog
eform

Generalized linear models                         Number of obs   =        111
Optimization     : ML                             Residual df     =        107
                                                  Scale parameter =          1
Deviance         =  57.99576103                   (1/df) Deviance =   .5420165
Pearson          =  105.1992692                   (1/df) Pearson  =   .9831707

Variance function: V(u) = u*(1-u/1)               [Binomial]
Link function    : g(u) = ln(u)                   [Log]

                                                  AIC             =   .5945564
Log likelihood   = -28.99788052                   BIC             =   -445.924

------------------------------------------------------------------------------------------
                            |                 OIM
      Adverseoutcomesupto2y | Risk Ratio   Std. Err.      z    P>|z|     [95% Conf. Interval]
----------------------------+-------------------------------------------------------------
                   CongZIKA |   6.639172   3.681354     3.41   0.001     2.239398    19.68324
Maternalinfectioninthe1sttr |   1.912284   1.010029     1.23   0.220     .6791498    5.384424
             Modeofdelivery |   .6487064   .5848379    -0.48   0.631     .1108286    3.797034
                      _cons |   .0457759   .0214718    -6.57   0.000     .0182545    .1147899
------------------------------------------------------------------------------------------
Note: _cons estimates baseline risk.

. lrtest A

Likelihood-ratio test                                 LR chi2(1)  =      0.29
(Assumption: A nested in .)                           Prob > chi2 =    0.5926

.
end of do-file

. do "C:\Users\lpomar\AppData\Local\Temp\STD3ed0_000000.tmp"

. glm Adverseoutcomesupto2y CongZIKA Maternalinfectioninthe1sttr Prematurity, fam(bin) link(log) nolog eform

Generalized linear models                         Number of obs   =        111
Optimization     : ML                             Residual df     =        107
                                                  Scale parameter =          1
```

```
Deviance         =  56.88109024                  (1/df) Deviance =    .531599
Pearson          =  104.0412757                  (1/df) Pearson  =   .9723484

Variance function: V(u) = u*(1-u/1)              [Binomial]
Link function    : g(u) = ln(u)                  [Log]

                                                 AIC             =   .5845143
Log likelihood   = -28.44054512                  BIC             =  -447.0386

------------------------------------------------------------------------------------------
                         |                 OIM
     Adverseoutcomesupto2y | Risk Ratio   Std. Err.      z    P>|z|     [95% Conf. Interval]
----------------------------+-------------------------------------------------------------
                 CongZIKA |   7.666233   4.622367     3.38   0.001      2.35152    24.99282
Maternalinfectioninthe1sttr |   1.923627   1.057265     1.19   0.234     .6550628    5.648833
           Prematurity37wg |   4.495911   4.734269     1.43   0.153     .5708058    35.41172
                    _cons |   .0370707   .0194581    -6.28   0.000     .0132508    .1037096
------------------------------------------------------------------------------------------
Note: _cons estimates baseline risk.

. lrtest A

Likelihood-ratio test                            LR chi2(1)  =      1.40
(Assumption: A nested in .)                      Prob > chi2 =    0.2366

.
end of do-file

. do "C:\Users\lpomar\AppData\Local\Temp\STD3ed0_000000.tmp"

.                 * Sub-analysis
. tab Adverseoutcomesupto2y CongZIKA if Structural==1

   Adverse |
  outcomes |       CongZIKA
  up to 2y |         0          1 |     Total
-----------+----------------------+----------
         0 |         1          1 |         2
         1 |         2          4 |         6
-----------+----------------------+----------
     Total |         3          5 |         8

. glm Adverseoutcomesupto2y CongZIKA if Structural==1, fam(bin) link(log) nolog eform

Generalized linear models                        Number of obs   =          8
Optimization     : ML                            Residual df     =          6
                                                 Scale parameter =          1
Deviance         =  8.823109245                  (1/df) Deviance =   1.470518
Pearson          =            8                  (1/df) Pearson  =   1.333333

Variance function: V(u) = u*(1-u/1)              [Binomial]
Link function    : g(u) = ln(u)                  [Log]

                                                 AIC             =   1.602889
Log likelihood   = -4.411554623                  BIC             =   -3.65354

------------------------------------------------------------------------------------
```

```
                     |               OIM
Adverseoutcomesupto2y | Risk Ratio   Std. Err.      z    P>|z|     [95% Conf. Interval]
----------------------+----------------------------------------------------------------
            CongZIKA |        1.2   .5585696     0.39   0.695     .4819128    2.988092
               _cons |   .6666667   .2721655    -0.99   0.321     .2995071    1.483919
---------------------------------------------------------------------------------------
Note: _cons estimates baseline risk.

. glm Adverseoutcomesupto2y CongZIKA Maternalinfectioninthe1sttr if Structural==1, fam(bin) link(log) nolog
eform
convergence not achieved

Generalized linear models                         Number of obs   =          8
Optimization     : ML                             Residual df     =          6
                                                  Scale parameter =          1
Deviance         =  8.823109245                   (1/df) Deviance =   1.470518
Pearson          =  7.988612804                   (1/df) Pearson  =   1.331435

Variance function: V(u) = u*(1-u/1)               [Binomial]
Link function    : g(u) = ln(u)                   [Log]

                                                  AIC             =   1.602889
Log likelihood   = -4.411554623                   BIC             =   -3.65354

---------------------------------------------------------------------------------------------
                           |               OIM
      Adverseoutcomesupto2y | Risk Ratio   Std. Err.      z    P>|z|     [95% Conf. Interval]
----------------------------+----------------------------------------------------------------
                  CongZIKA |   1.095445    .509902     0.20   0.845     .4399242    2.727743
Maternalinfectioninthe1sttr |   1.095445          .        .       .            .           .
                     _cons |   .6666667   .2721655    -0.99   0.321     .2995071    1.483919
---------------------------------------------------------------------------------------------
Note: _cons estimates baseline risk.
Warning: convergence not achieved


.
. tab Adverseoutcomesupto2y CongZIKA if Structural==0

   Adverse |
  outcomes |       CongZIKA
  up to 2y |         0          1 |     Total
-----------+----------------------+----------
         0 |        90          8 |        98 
         1 |         3          2 |         5 
-----------+----------------------+----------
     Total |        93         10 |       103 

. glm Adverseoutcomesupto2y CongZIKA if Structural==0, fam(bin) link(log) nolog eform

Generalized linear models                         Number of obs   =        103
Optimization     : ML                             Residual df     =        101
                                                  Scale parameter =          1
Deviance         =  36.51413981                   (1/df) Deviance =   .3615261
Pearson          =  102.9999995                   (1/df) Pearson  =   1.019802

Variance function: V(u) = u*(1-u/1)               [Binomial]
Link function    : g(u) = ln(u)                   [Log]
```

```
                                  AIC             =  .3933412
Log likelihood  =  -18.2570699                BIC             =  -431.5935

------------------------------------------------------------------------------
                 |                 OIM
Adverseoutcomesupto2y | Risk Ratio   Std. Err.      z    P>|z|     [95% Conf. Interval]
---------------------+----------------------------------------------------------------
        CongZIKA |        6.2   5.270294     2.15   0.032     1.171737    32.80601
           _cons |   .0322581   .0183213    -6.05   0.000     .0105971    .0981948
------------------------------------------------------------------------------
Note: _cons estimates baseline risk.

. glm Adverseoutcomesupto2y CongZIKA Maternalinfectioninthe1sttr if Structural==0, fam(bin) link(log) nolog
eform

Generalized linear models                          Number of obs   =        103
Optimization     : ML                              Residual df     =        100
                                                   Scale parameter =          1
Deviance         =  36.50351232                    (1/df) Deviance =   .3650351
Pearson          =  104.3085701                    (1/df) Pearson  =   1.043086

Variance function: V(u) = u*(1-u/1)                [Binomial]
Link function    : g(u) = ln(u)                    [Log]

                                                   AIC             =  .4126555
Log likelihood   = -18.25175616                    BIC             =  -426.9694

-------------------------------------------------------------------------------------
                       |                 OIM
    Adverseoutcomesupto2y | Risk Ratio   Std. Err.      z    P>|z|     [95% Conf. Interval]
---------------------------+----------------------------------------------------------------
               CongZIKA |   6.229669   5.300758     2.15   0.032     1.175403    33.01743
Maternalinfectioninthe1sttr |   .8979346   .9501202    -0.10   0.919     .1128687     7.14358
                  _cons |   .0329284   .0197506    -5.69   0.000     .0101628    .1066905
-------------------------------------------------------------------------------------
Note: _cons estimates baseline risk.

.
. tab Neurologicimpairments CongZIKA, freq

Neurologic |
impairment |       CongZIKA
         s |         0          1 |     Total
-----------+----------------------+----------
         0 |        92         10 |       102
         1 |         4          5 |         9
-----------+----------------------+----------
     Total |        96         15 |       111

. glm Neurologicimpairments CongZIKA, fam(bin) link(log) nolog eform

Generalized linear models                          Number of obs   =        111
Optimization     : ML                              Residual df     =        109
                                                   Scale parameter =          1
Deviance         =  52.35082474                    (1/df) Deviance =   .4802828
Pearson          =          111                    (1/df) Pearson  =   1.018349
```

```
Variance function: V(u) = u*(1-u/1)            [Binomial]
Link function    : g(u) = ln(u)                [Log]

                                        AIC             =  .5076651
Log likelihood   = -26.17541237                 BIC             =  -460.988

------------------------------------------------------------------------------
                  |                 OIM
Neurologicimpairments | Risk Ratio   Std. Err.      z    P>|z|     [95% Conf. Interval]
---------------------+--------------------------------------------------------
          CongZIKA |          8   4.885352     3.41   0.001     2.417061    26.47844
             _cons |   .0416667   .0203947    -6.49   0.000     .0159643    .1087498
------------------------------------------------------------------------------
Note: _cons estimates baseline risk.

. glm Neurologicimpairments CongZIKA Maternalinfectioninthe1sttr, fam(bin) link(log) nolog eform

Generalized linear models                          Number of obs   =        111
Optimization     : ML                              Residual df     =        108
                                                   Scale parameter =          1
Deviance         =  51.25680245                    (1/df) Deviance =      .4746
Pearson          =  114.8890414                    (1/df) Pearson  =   1.063787

Variance function: V(u) = u*(1-u/1)            [Binomial]
Link function    : g(u) = ln(u)                [Log]

                                        AIC             =   .515827
Log likelihood   = -25.62840122                 BIC             =  -457.3725

----------------------------------------------------------------------------------
                      |                 OIM
     Neurologicimpairments | Risk Ratio   Std. Err.      z    P>|z|     [95% Conf. Interval]
----------------------------+-----------------------------------------------------
               CongZIKA |   7.129378   4.438435     3.16   0.002     2.104414    24.15305
Maternalinfectioninthe1sttr |   1.875192    1.10714     1.06   0.287     .5895002    5.964958
                  _cons |   .0351488   .0185808    -6.33   0.000      .012472    .0990567
----------------------------------------------------------------------------------
Note: _cons estimates baseline risk.


.
. tab Delayinmotoracquisitions CongZIKA, freq

  Delay in |
     motor |
acquisitio |       CongZIKA
        ns |         0          1 |     Total
-----------+----------------------+----------
         0 |        95         13 |       108
         1 |         1          2 |         3
-----------+----------------------+----------
     Total |        96         15 |       111

. glm Delayinmotoracquisitions CongZIKA, fam(bin) link(log) nolog eform

Generalized linear models                          Number of obs   =        111
Optimization     : ML                              Residual df     =        109
                                                   Scale parameter =          1
```

```
Deviance        =  22.89847737                  (1/df) Deviance =   .2100778
Pearson         =         111                  (1/df) Pearson  =   1.018349

Variance function: V(u) = u*(1-u/1)              [Binomial]
Link function    : g(u) = ln(u)                  [Log]

                                             AIC             =  .2423286
Log likelihood   = -11.44923869                  BIC             =  -490.4403

------------------------------------------------------------------------------
                         |                 OIM
Delayinmotoracquisitions | Risk Ratio   Std. Err.      z    P>|z|     [95% Conf. Interval]
-------------------------+----------------------------------------------------------------
                CongZIKA |       12.8   15.26862     2.14   0.033     1.235507    132.6096
                   _cons |   .0104167   .0103623    -4.59   0.000     .0014824    .0731957
------------------------------------------------------------------------------
Note: _cons estimates baseline risk.

. glm Delayinmotoracquisitions CongZIKA Maternalinfectioninthe1sttr, fam(bin) link(log) nolog eform

Generalized linear models                          Number of obs   =        111
Optimization     : ML                              Residual df     =        108
                                                   Scale parameter =          1
Deviance         =  21.50365408                    (1/df) Deviance =   .1991079
Pearson          =  60.33332903                    (1/df) Pearson  =   .5586419

Variance function: V(u) = u*(1-u/1)              [Binomial]
Link function    : g(u) = ln(u)                  [Log]

                                             AIC             =  .2477807
Log likelihood   = -10.75182704                  BIC             =  -487.1256

---------------------------------------------------------------------------------
                            |                 OIM
   Delayinmotoracquisitions | Risk Ratio   Std. Err.      z    P>|z|     [95% Conf. Interval]
----------------------------+----------------------------------------------------------------
                   CongZIKA |   9.000001   11.03903     1.79   0.073     .8131929    99.60738
Maternalinfectioninthe1sttr |   3.923077    4.78711     1.12   0.263     .3588837    42.88447
                      _cons |   .0065359   .0076315    -4.31   0.000     .0006629    .0644446
---------------------------------------------------------------------------------
Note: _cons estimates baseline risk.


.
. tab Neurosensoryalterations CongZIKA, freq

Neurosenso |
        ry |
alteration |       CongZIKA
         s |         0          1 |     Total
-----------+----------------------+----------
         0 |        95         12 |       107
         1 |         1          3 |         4
-----------+----------------------+----------
     Total |        96         15 |       111

. glm Neurosensoryalterations CongZIKA, fam(bin) link(log) nolog eform
```

```
Generalized linear models                          Number of obs   =        111
Optimization     : ML                              Residual df     =        109
                                                   Scale parameter =          1
Deviance         =  26.13031606                    (1/df) Deviance =   .2397277
Pearson          =  110.9999985                    (1/df) Pearson  =   1.018349

Variance function: V(u) = u*(1-u/1)                [Binomial]
Link function    : g(u) = ln(u)                    [Log]

                                                   AIC             =   .2714443
Log likelihood   = -13.06515803                    BIC             =  -487.2085

------------------------------------------------------------------------------
                     |                 OIM
Neurosensoryalterations | Risk Ratio   Std. Err.      z    P>|z|     [95% Conf. Interval]
---------------------+--------------------------------------------------------
            CongZIKA |       19.2   21.51985     2.64   0.008     2.134287    172.7228
               _cons |   .0104167   .0103623    -4.59   0.000     .0014824    .0731957
------------------------------------------------------------------------------
Note: _cons estimates baseline risk.

. glm Neurosensoryalterations CongZIKA Maternalinfectioninthe1sttr, fam(bin) link(log) nolog eform

Generalized linear models                          Number of obs   =        111
Optimization     : ML                              Residual df     =        108
                                                   Scale parameter =          1
Deviance         =  25.66113713                    (1/df) Deviance =   .2376031
Pearson          =  185.4801619                    (1/df) Pearson  =   1.717409

Variance function: V(u) = u*(1-u/1)                [Binomial]
Link function    : g(u) = ln(u)                    [Log]

                                                   AIC             =   .2852355
Log likelihood   = -12.83056856                    BIC             =  -482.9681

---------------------------------------------------------------------------------
                        |                 OIM
   Neurosensoryalterations | Risk Ratio   Std. Err.      z    P>|z|     [95% Conf. Interval]
------------------------+--------------------------------------------------------
               CongZIKA |   22.47253   25.37779     2.76   0.006      2.45701    205.5404
Maternalinfectioninthe1sttr |   .4965815   .5383341    -0.65   0.518     .0593226    4.156819
                  _cons |   .0115768   .0115947    -4.45   0.000     .0016258     .082434
---------------------------------------------------------------------------------
Note: _cons estimates baseline risk.


.
. cs Adverseoutcomesupto2y CongZIKA, by(Maternalinfectioninthe1sttr)

Maternal infecti |       RR       [95% Conf. Interval]   M-H Weight
-----------------+-------------------------------------------------
               0 |   8.444444     1.993798   35.76523     .3176471
               1 |          5     1.072846   23.30251     .4615385
-----------------+-------------------------------------------------
           Crude |       7.68     2.674882   22.05047
    M-H combined |   6.404181      2.23786   18.32712
-------------------------------------------------------------------
Test of homogeneity (M-H)      chi2(1) =    0.240  Pr>chi2 = 0.6239
```

```
.
end of do-file

. do "C:\Users\lpomar\AppData\Local\Temp\STD3ed0_000000.tmp"

.
. ** Adverse outcomes at 3years of life:
. tab Neurodevelopment2SDat3y CongZIKA

Neurodevel |
   opment |
 <-2SD at |       CongZIKA
       3y |         0          1 |     Total
-----------+----------------------+----------
         0 |        44          4 |        48
         1 |         7          7 |        14
-----------+----------------------+----------
     Total |        51         11 |        62

. glm Neurodevelopment2SDat3y CongZIKA, fam(bin) link(log) nolog eform

Generalized linear models                         Number of obs   =         62
Optimization     : ML                             Residual df     =         60
                                                  Scale parameter =          1
Deviance         =  55.21538369                   (1/df) Deviance =   .9202564
Pearson          =  61.99999999                   (1/df) Pearson  =   1.033333

Variance function: V(u) = u*(1-u/1)               [Binomial]
Link function    : g(u) = ln(u)                   [Log]

                                                  AIC             =   .9550868
Log likelihood   = -27.60769185                   BIC             =  -192.4127

------------------------------------------------------------------------------------------
                        |                 OIM
Neurodevelopment2SDat3y | Risk Ratio   Std. Err.      z    P>|z|     [95% Conf. Interval]
------------------------+-----------------------------------------------------------------
               CongZIKA |   4.636364   1.940623     3.66   0.000     2.041238     10.5308
                  _cons |   .1372549   .0481859    -5.66   0.000     .0689759    .2731229
------------------------------------------------------------------------------------------
Note: _cons estimates baseline risk.

. est store CRUDE

.
.         * Adjusted on maternal infection in the 1st trimester
. glm Neurodevelopment2SDat3y CongZIKA Maternalinfectioninthe1sttr, fam(bin) link(log) nolog eform

Generalized linear models                         Number of obs   =         62
Optimization     : ML                             Residual df     =         59
                                                  Scale parameter =          1
Deviance         =  54.33232261                   (1/df) Deviance =   .9208868
Pearson          =  61.13050756                   (1/df) Pearson  =    1.03611

Variance function: V(u) = u*(1-u/1)               [Binomial]
Link function    : g(u) = ln(u)                   [Log]
```

```
                                   AIC             =   .973102
Log likelihood   = -27.16616131                  BIC             =  -189.1686

------------------------------------------------------------------------------
                            |                 OIM
   Neurodevelopment2SDat3y | Risk Ratio   Std. Err.      z    P>|z|     [95% Conf. Interval]
----------------------------+----------------------------------------------------------------
               CongZIKA |   4.432214   1.867274     3.53   0.000     1.940943    10.12112
Maternalinfectioninthe1sttr |   1.431828   .5386402     0.95   0.340     .6849787    2.992987
                  _cons |   .1222047   .0461757    -5.56   0.000     .0582716    .2562823
------------------------------------------------------------------------------
Note: _cons estimates baseline risk.

. est store A

. lrtest CRUDE

Likelihood-ratio test                                 LR chi2(1)  =      0.88
(Assumption: CRUDE nested in A)                       Prob > chi2 =    0.3474

.
.         * Effect modifiers?
. glm Neurodevelopment2SDat3y CongZIKA Maternalinfectioninthe1sttr Maternalage, fam(bin) link(log) nolog
eform

Generalized linear models                          Number of obs   =         62
Optimization     : ML                              Residual df     =         58
                                                   Scale parameter =          1
Deviance         =  54.30076072                    (1/df) Deviance =     .93622
Pearson          =  60.23498069                    (1/df) Pearson  =   1.038534

Variance function: V(u) = u*(1-u/1)                [Binomial]
Link function    : g(u) = ln(u)                    [Log]

                                                   AIC             =   1.004851
Log likelihood   = -27.15038036                    BIC             =   -185.073

------------------------------------------------------------------------------
                            |                 OIM
   Neurodevelopment2SDat3y | Risk Ratio   Std. Err.      z    P>|z|     [95% Conf. Interval]
----------------------------+----------------------------------------------------------------
               CongZIKA |   4.362711   1.877119     3.42   0.001     1.877219    10.13907
Maternalinfectioninthe1sttr |   1.467104    .593885     0.95   0.344     .6635809    3.243606
   Maternalageatbirthyears |   1.005938   .0337233     0.18   0.860     .9419665    1.074255
                  _cons |   .1033932   .1065144    -2.20   0.028     .0137276    .7787333
------------------------------------------------------------------------------
Note: _cons estimates baseline risk.

. lrtest A

Likelihood-ratio test                                 LR chi2(1)  =      0.03
(Assumption: A nested in .)                           Prob > chi2 =    0.8590

. glm Neurodevelopment2SDat3y CongZIKA Maternalinfectioninthe1sttr Low, fam(bin) link(log) nolog eform
convergence not achieved

Generalized linear models                          Number of obs   =         60
```

```
Optimization     : ML                           Residual df     =         56
                                     Scale parameter =          1
Deviance         =  52.70108623                 (1/df) Deviance =   .9410908
Pearson          =  61.34982158                 (1/df) Pearson  =   1.095533

Variance function: V(u) = u*(1-u/1)             [Binomial]
Link function    : g(u) = ln(u)                 [Log]

                                     AIC             =   1.011685
Log likelihood   = -26.35054311                 BIC             =  -176.5822

------------------------------------------------------------------------------------------
                          |                 OIM
   Neurodevelopment2SDat3y | Risk Ratio   Std. Err.      z    P>|z|     [95% Conf. Interval]
---------------------------+--------------------------------------------------------------
                 CongZIKA |   4.959629   2.066594     3.84   0.000     2.191633    11.22356
Maternalinfectioninthe1sttr |   1.892683   .8958861     1.35   0.178       .74846     4.78616
    Lowsocioeconomicstatus |   1.879118   .8997054     1.32   0.188     .7352012    4.802882
                    _cons |   .0784325   .0461682    -4.32   0.000     .0247427    .2486247
------------------------------------------------------------------------------------------
Note: _cons estimates baseline risk.
Warning: parameter estimates produce inadmissible mean estimates in one or
         more observations.
Warning: convergence not achieved

. lrtest A
observations differ: 60 vs. 62
r(498);

end of do-file

r(498);

. do "C:\Users\lpomar\AppData\Local\Temp\STD3ed0_000000.tmp"

. glm Neurodevelopment2SDat3y CongZIKA Maternalinfectioninthe1sttr Alcohol, fam(bin) link(log) nolog eform
convergence not achieved

Generalized linear models                        Number of obs   =         62
Optimization     : ML                           Residual df     =         60
                                     Scale parameter =          1
Deviance         =  97.87058364                 (1/df) Deviance =   1.631176
Pearson          =  100000055.6                 (1/df) Pearson  =    1666668

Variance function: V(u) = u*(1-u/1)             [Binomial]
Link function    : g(u) = ln(u)                 [Log]

                                     AIC             =   1.643074
Log likelihood   = -48.93529182                 BIC             =  -149.7575

------------------------------------------------------------------------------------------
                          |                 OIM
   Neurodevelopment2SDat3y | Risk Ratio   Std. Err.      z    P>|z|     [95% Conf. Interval]
---------------------------+--------------------------------------------------------------
                 CongZIKA |   2.139565          .        .       .            .           .
Maternalinfectioninthe1sttr |   1.265906          .        .       .            .           .
                  Alcohol |   .1674685   .1603071    -1.87   0.062     .0256526     1.09329
```

```
                     _cons |   .3858783   3.86e-09 -9.5e+07   0.000     .3858783    .3858783
------------------------------------------------------------------------------
Note: _cons estimates baseline risk.
Warning: parameter estimates produce inadmissible mean estimates in one or
         more observations.
Warning: convergence not achieved


.
end of do-file

. tab Neurodevelopment2SDat3y CongZIKA if Alcohol==1

Neurodevel |
    opment |
  <-2SD at |       CongZIKA
        3y |         0          1 |     Total
-----------+----------------------+----------
         0 |        11          1 |        12
         1 |         1          0 |         1
-----------+----------------------+----------
     Total |        12          1 |        13

. tab Neurodevelopment2SDat3y CongZIKA if Alcohol==0

Neurodevel |
    opment |
  <-2SD at |       CongZIKA
        3y |         0          1 |     Total
-----------+----------------------+----------
         0 |        33          3 |        36
         1 |         6          7 |        13
-----------+----------------------+----------
     Total |        39         10 |        49

. do "C:\Users\lpomar\AppData\Local\Temp\STD3ed0_000000.tmp"

. glm Neurodevelopment2SDat3y CongZIKA Maternalinfectioninthe1sttr Smoking, fam(bin) link(log) nolog eform
note: Smoking != 0 predicts failure perfectly
      Smoking dropped and 2 obs not used



Generalized linear models                         Number of obs   =         59
Optimization     : ML                             Residual df     =         58
                                                  Scale parameter =          1
Deviance         =  54.08660189                   (1/df) Deviance =   .9325276
Pearson          =  58.53110455                   (1/df) Pearson  =   1.009157

Variance function: V(u) = u*(1-u/1)               [Binomial]
Link function    : g(u) = ln(u)                   [Log]

                                                  AIC             =   .9736936
Log likelihood   = -26.69765599                   BIC             =  -184.3441

------------------------------------------------------------------------------
                           |                 OIM
   Neurodevelopment2SDat3y | Risk Ratio   Std. Err.      z    P>|z|     [95% Conf. Interval]
---------------------------+--------------------------------------------------------------
```

```
              CongZIKA |    4.16078   1.748102     3.39   0.001     1.826218    9.479752
Maternalinfectioninthe1sttr |   1.437647    .540676     0.97   0.334     .6879061    3.004523
               Smoking |          1  (omitted)
                 _cons |   .1298248   .0487985    -5.43   0.000     .0621453    .2712106
------------------------------------------------------------------------------------------
Note: _cons estimates baseline risk.

. lrtest A
df(unrestricted) = df(restricted) = 3
r(498);

end of do-file

r(498);

. do "C:\Users\lpomar\AppData\Local\Temp\STD3ed0_000000.tmp"

. glm Neurodevelopment2SDat3y CongZIKA Maternalinfectioninthe1sttr Leadp, fam(bin) link(log) nolog eform
convergence not achieved

Generalized linear models                          Number of obs   =         62
Optimization     : ML                              Residual df     =         60
                                                   Scale parameter =          1
Deviance         =  100.2234335                    (1/df) Deviance =   1.670391
Pearson          =  100000046.9                    (1/df) Pearson  =    1666667

Variance function: V(u) = u*(1-u/1)                [Binomial]
Link function    : g(u) = ln(u)                    [Log]

                                                   AIC             =   1.681023
Log likelihood   = -50.11171674                    BIC             =  -147.4046

------------------------------------------------------------------------------------------
                        |                 OIM
   Neurodevelopment2SDat3y | Risk Ratio   Std. Err.      z    P>|z|     [95% Conf. Interval]
---------------------------+--------------------------------------------------------------
              CongZIKA |   2.495347          .        .        .            .           .
Maternalinfectioninthe1sttr |    1.42363          .        .        .            .           .
         Leadpoisoning |   1.066297    .870057     0.08   0.937     .2154423    5.277468
                 _cons |   .3128134   3.13e-09 -1.2e+08   0.000     .3128134    .3128134
------------------------------------------------------------------------------------------
Note: _cons estimates baseline risk.
Warning: parameter estimates produce inadmissible mean estimates in one or
         more observations.
Warning: convergence not achieved

. lrtest A

Likelihood-ratio test                                 LR chi2(1)  =     45.89
(Assumption: . nested in A)                           Prob > chi2 =    0.0000

.
end of do-file

. tab Neurodevelopment2SDat3y CongZIKA if Leadp==0

Neurodevel |
```

```
   opment |
  <-2SD at |       CongZIKA
        3y |         0          1 |     Total
-----------+----------------------+----------
         0 |        42          4 |        46
         1 |         6          7 |        13
-----------+----------------------+----------
     Total |        48         11 |        59

. tab Neurodevelopment2SDat3y CongZIKA if Leadp==1

Neurodevel |
   opment |
  <-2SD at |  CongZIKA
        3y |         0 |     Total
-----------+-----------+----------
         0 |         2 |         2
         1 |         1 |         1
-----------+-----------+----------
     Total |         3 |         3

. do "C:\Users\lpomar\AppData\Local\Temp\STD3ed0_000000.tmp"

. glm Neurodevelopment2SDat3y CongZIKA Maternalinfectioninthe1sttr Anymaternalco, fam(bin) link(log) nolog
eform
note: Anymaternalcomorbidities != 0 predicts failure perfectly
      Anymaternalcomorbidities dropped and 11 obs not used

--Break--
r(1);

end of do-file

--Break--
r(1);

. tab Neurodevelopment2SDat3y CongZIKA if Anynaternal==1
Anynaternal not found
r(111);

. tab Neurodevelopment2SDat3y CongZIKA if Anymaternal==1

Neurodevel |
   opment |
  <-2SD at |       CongZIKA
        3y |         0          1 |     Total
-----------+----------------------+----------
         0 |         8          3 |        11
-----------+----------------------+----------
     Total |         8          3 |        11

. tab Neurodevelopment2SDat3y CongZIKA if Anymaternal==0

Neurodevel |
   opment |
  <-2SD at |       CongZIKA
        3y |         0          1 |     Total
```

```
-----------+----------------------+----------
         0 |        36          1 |        37 
         1 |         7          7 |        14 
-----------+----------------------+----------
     Total |        43          8 |        51 

. do "C:\Users\lpomar\AppData\Local\Temp\STD3ed0_000000.tmp"

. glm Neurodevelopment2SDat3y CongZIKA Maternalinfectioninthe1sttr Infantgender, fam(bin) link(log) nolog
eform
convergence not achieved

Generalized linear models                          Number of obs   =         62
Optimization     : ML                              Residual df     =         60
                                                   Scale parameter =          1
Deviance         =  90.4375652                     (1/df) Deviance =   1.507293
Pearson          =  100000047.9                    (1/df) Pearson  =    1666667

Variance function: V(u) = u*(1-u/1)                [Binomial]
Link function    : g(u) = ln(u)                    [Log]

                                                   AIC             =   1.523187
Log likelihood   =  -45.2187826                    BIC             =  -157.1905

---------------------------------------------------------------------------------------------
                            |                 OIM
    Neurodevelopment2SDat3y | Risk Ratio   Std. Err.      z    P>|z|     [95% Conf. Interval]
----------------------------+----------------------------------------------------------------
                   CongZIKA |   2.135364          .        .       .            .           .
Maternalinfectioninthe1sttr |   1.352287          .        .       .            .           .
               Infantgender |   .3170819   .1175789    -3.10   0.002     .1532967    .6558587
                      _cons |   .4065965   4.07e-09 -9.0e+07   0.000     .4065965    .4065965
---------------------------------------------------------------------------------------------
Note: _cons estimates baseline risk.
Warning: parameter estimates produce inadmissible mean estimates in one or
         more observations.
Warning: convergence not achieved

. lrtest A

Likelihood-ratio test                                 LR chi2(1)  =     36.11
(Assumption: . nested in A)                           Prob > chi2 =    0.0000

. 
end of do-file

. tab Neurodevelopment2SDat3y Infantgender, chi2

Neurodevel |
    opment |
  <-2SD at |     Infant gender
        3y |         0          1 |     Total
-----------+----------------------+----------
         0 |        16         32 |        48 
         1 |         8          6 |        14 
-----------+----------------------+----------
     Total |        24         38 |        62 
```

```
         Pearson chi2(1) =   2.5898   Pr = 0.108

. glm Neurodevelopment2SDat3y CongZIKA Infantgender, fam(bin) link(log) nolog eform

Generalized linear models                          Number of obs   =         62
Optimization     : ML                              Residual df     =         59
                                                   Scale parameter =          1
Deviance         =    52.950206                    (1/df) Deviance =   .8974611
Pearson          =  63.68186783                    (1/df) Pearson  =   1.079354

Variance function: V(u) = u*(1-u/1)                [Binomial]
Link function    : g(u) = ln(u)                    [Log]

                                                   AIC             =   .9508098
Log likelihood   =   -26.475103                    BIC             =  -190.5507

------------------------------------------------------------------------------------------
                        |                 OIM
Neurodevelopment2SDat3y | Risk Ratio   Std. Err.      z    P>|z|     [95% Conf. Interval]
------------------------+-----------------------------------------------------------------
               CongZIKA |   4.286504   1.776361     3.51   0.000     1.902648    9.657126
           Infantgender |   .5602415   .2291838    -1.42   0.157     .2512843    1.249065
                  _cons |   .1906202   .0758524    -4.17   0.000     .0873887    .4157981
------------------------------------------------------------------------------------------
Note: _cons estimates baseline risk.

. lrtest A
df(unrestricted) = df(restricted) = 3
r(498);

. glm Neurodevelopment2SDat3y CongZIKA Twins, fam(bin) link(log) nolog eform
note: Twins != 0 predicts failure perfectly
      Twins dropped and 3 obs not used


Generalized linear models                          Number of obs   =         59
Optimization     : ML                              Residual df     =         60
                                                   Scale parameter =          1
Deviance         =  55.24601818                    (1/df) Deviance =    .920767
Pearson          =  59.51219511                    (1/df) Pearson  =   .9918699

Variance function: V(u) = u*(1-u/1)                [Binomial]
Link function    : g(u) = ln(u)                    [Log]

                                                   AIC             =   .9403265
Log likelihood   = -27.15012226                    BIC             =   -192.382

------------------------------------------------------------------------------------------
                        |                 OIM
Neurodevelopment2SDat3y | Risk Ratio   Std. Err.      z    P>|z|     [95% Conf. Interval]
------------------------+-----------------------------------------------------------------
               CongZIKA |   4.363636    1.82007     3.53   0.000     1.926695    9.882894
                  Twins |          1  (omitted)
                  _cons |   .1458333   .0509424    -5.51   0.000     .0735387    .2891996
------------------------------------------------------------------------------------------
Note: _cons estimates baseline risk.
```

```
.
. lrtest A
observations differ: 62 vs. 59
r(498);

. glm Neurodevelopment2SDat3y CongZIKA Modeofdelivery, fam(bin) link(log) nolog eform
--Break--
r(1);


.
. lrtest A
df(unrestricted) = df(restricted) = 3
r(498);

. tab Neurodevelopment2SDat3y CongZIKA Modeofdelivery
too many variables specified
r(103);

. tab Neurodevelopment2SDat3y CongZIKA if Modeofdeliver==1

Neurodevel |
    opment |
  <-2SD at |       CongZIKA
        3y |         0          1 |     Total
-----------+----------------------+----------
         0 |         6          0 |         6
         1 |         1          1 |         2
-----------+----------------------+----------
     Total |         7          1 |         8

. tab Neurodevelopment2SDat3y CongZIKA if Modeofdeliver==0

Neurodevel |
    opment |
  <-2SD at |       CongZIKA
        3y |         0          1 |     Total
-----------+----------------------+----------
         0 |        38          4 |        42
         1 |         6          6 |        12
-----------+----------------------+----------
     Total |        44         10 |        54

. do "C:\Users\lpomar\AppData\Local\Temp\STD3ed0_000000.tmp"

. glm Neurodevelopment2SDat3y CongZIKA Maternalinfectioninthe1sttr Prematurity, fam(bin) link(log) nolog
eform
--Break--
r(1);

end of do-file

--Break--
r(1);

. glm Neurodevelopment2SDat3y CongZIKA Prematurity, fam(bin) link(log) nolog eform

Generalized linear models                          Number of obs   =         62
```

```
Optimization     : ML                            Residual df     =         59
                                                  Scale parameter =          1
Deviance         =  54.40961952                  (1/df) Deviance =   .9221969
Pearson          =  61.99999999                  (1/df) Pearson  =   1.050847

Variance function: V(u) = u*(1-u/1)              [Binomial]
Link function    : g(u) = ln(u)                  [Log]

                                                  AIC             =   .9743487
Log likelihood   = -27.20480976                  BIC             =  -189.0913

------------------------------------------------------------------------------------
                        |                 OIM
Neurodevelopment2SDat3y | Risk Ratio   Std. Err.      z    P>|z|     [95% Conf. Interval]
------------------------+-----------------------------------------------------------
               CongZIKA |   5.090909   2.264061     3.66   0.000     2.129334    12.17158
        Prematurity37wg |   2.666667   2.403701     1.09   0.277     .4557283    15.60384
                  _cons |       .125   .0477352    -5.45   0.000      .059136    .2642214
------------------------------------------------------------------------------------
Note: _cons estimates baseline risk.

. do "C:\Users\lpomar\AppData\Local\Temp\STD3ed0_000000.tmp"

. lrtest A
df(unrestricted) = df(restricted) = 3
r(498);

end of do-file

r(498);

. do "C:\Users\lpomar\AppData\Local\Temp\STD3ed0_000000.tmp"

.                 * Sub-analysis
. tab Neurodevelopment2SDat3y CongZIKA if Structural==1, exact

Neurodevel |
    opment |
  <-2SD at |       CongZIKA
        3y |         0          1 |     Total
-----------+----------------------+----------
         0 |         2          0 |         2
         1 |         0          4 |         4
-----------+----------------------+----------
     Total |         2          4 |         6

           Fisher's exact =                 0.067
   1-sided Fisher's exact =                 0.067

. tab Neurodevelopment2SDat3y CongZIKA if Structural==0

Neurodevel |
    opment |
  <-2SD at |       CongZIKA
        3y |         0          1 |     Total
-----------+----------------------+----------
         0 |        42          4 |        46
```

```
        1 |         7          3 |        10
-----------+----------------------+----------
     Total |        49          7 |        56

. glm Neurodevelopment2SDat3y CongZIKA if Structural==0, fam(bin) link(log) nolog eform

Generalized linear models                          Number of obs   =         56
Optimization     : ML                              Residual df     =         54
                                                   Scale parameter =          1
Deviance         =  49.75211266                    (1/df) Deviance =   .9213354
Pearson          =           56                    (1/df) Pearson  =   1.037037

Variance function: V(u) = u*(1-u/1)                [Binomial]
Link function    : g(u) = ln(u)                    [Log]

                                                   AIC             =   .9598592
Log likelihood   = -24.87605633                    BIC             =  -167.6169

------------------------------------------------------------------------------------------
                        |                 OIM
Neurodevelopment2SDat3y | Risk Ratio   Std. Err.      z    P>|z|     [95% Conf. Interval]
------------------------+-----------------------------------------------------------------
               CongZIKA |          3   1.678191     1.96   0.050     1.002216    8.980096
                  _cons |   .1428571   .0499896    -5.56   0.000     .0719521    .2836354
------------------------------------------------------------------------------------------
Note: _cons estimates baseline risk.

. glm Neurodevelopment2SDat3y CongZIKA Maternalinfectioninthe1sttr if Structural==0, fam(bin) link(log) nolog
eform

Generalized linear models                          Number of obs   =         56
Optimization     : ML                              Residual df     =         53
                                                   Scale parameter =          1
Deviance         =  49.68726937                    (1/df) Deviance =   .9374956
Pearson          =  55.37021041                    (1/df) Pearson  =   1.044721

Variance function: V(u) = u*(1-u/1)                [Binomial]
Link function    : g(u) = ln(u)                    [Log]

                                                   AIC             =   .9944155
Log likelihood   = -24.84363469                    BIC             =  -163.6564

----------------------------------------------------------------------------------------------
                            |                 OIM
    Neurodevelopment2SDat3y | Risk Ratio   Std. Err.      z    P>|z|     [95% Conf. Interval]
----------------------------+-----------------------------------------------------------------
                   CongZIKA |   2.996958   1.673399     1.97   0.049     1.003226    8.952877
Maternalinfectioninthe1sttr |   1.164735   .6840908     0.26   0.795     .3683713    3.682718
                      _cons |   .1368373   .0537176    -5.07   0.000     .0633946    .2953634
----------------------------------------------------------------------------------------------
Note: _cons estimates baseline risk.

.
. tab Motordomain CongZIKA, freq

     Motor |
    domain |       CongZIKA
```

```
    <-2SD |         0          1 |     Total
-----------+----------------------+----------
         0 |        50          9 |        59
         1 |         1          2 |         3
-----------+----------------------+----------
     Total |        51         11 |        62

. glm Motordomain CongZIKA, fam(bin) link(log) nolog eform

Generalized linear models                          Number of obs   =         62
Optimization     : ML                              Residual df     =         60
                                                   Scale parameter =          1
Deviance         =  20.27497888                    (1/df) Deviance =   .3379163
Pearson          =  61.99998564                    (1/df) Pearson  =   1.033333

Variance function: V(u) = u*(1-u/1)                [Binomial]
Link function    : g(u) = ln(u)                    [Log]

                                                   AIC             =   .3915319
Log likelihood   = -10.13748944                    BIC             =  -227.3531

------------------------------------------------------------------------------
             |                 OIM
Motordomain2SD | Risk Ratio   Std. Err.      z    P>|z|     [95% Conf. Interval]
---------------+--------------------------------------------------------------
    CongZIKA |   9.272729   10.93035     1.89   0.059     .9201159    93.44855
       _cons |   .0196078   .0194147    -3.97   0.000     .0028159    .1365353
------------------------------------------------------------------------------
Note: _cons estimates baseline risk.

. glm Motordomain CongZIKA Maternalinfectioninthe1sttr, fam(bin) link(log) nolog eform

Generalized linear models                          Number of obs   =         62
Optimization     : ML                              Residual df     =         59
                                                   Scale parameter =          1
Deviance         =  18.30323253                    (1/df) Deviance =   .3102243
Pearson          =  112.0075866                    (1/df) Pearson  =   1.898434

Variance function: V(u) = u*(1-u/1)                [Binomial]
Link function    : g(u) = ln(u)                    [Log]

                                                   AIC             =   .3919876
Log likelihood   = -9.151616264                    BIC             =  -225.1977

----------------------------------------------------------------------------------------
                         |                 OIM
           Motordomain2SD | Risk Ratio   Std. Err.      z    P>|z|     [95% Conf. Interval]
---------------------------+------------------------------------------------------------
                CongZIKA |   9.344466   10.71218     1.95   0.051     .9880204    88.37778
Maternalinfectioninthe1sttr |   4.573782   5.188736     1.34   0.180     .4950261    42.25935
                   _cons |   .0093458   .0116673    -3.74   0.000      .000809    .1079583
----------------------------------------------------------------------------------------
Note: _cons estimates baseline risk.

.
. tab Congitive CongZIKA, freq
```

```
 Congitive |
    domain |      CongZIKA
    <-2SD |        0          1 |     Total
-----------+----------------------+----------
         0 |        48          5 |        53
         1 |         3          6 |         9
-----------+----------------------+----------
     Total |        51         11 |        62

. glm Congitive CongZIKA, fam(bin) link(log) nolog eform

Generalized linear models                         Number of obs   =         62
Optimization     : ML                             Residual df     =         60
                                                  Scale parameter =          1
Deviance         =  37.97744701                   (1/df) Deviance =   .6329575
Pearson          =  61.99999999                   (1/df) Pearson  =   1.033333

Variance function: V(u) = u*(1-u/1)               [Binomial]
Link function    : g(u) = ln(u)                   [Log]

                                                  AIC             =   .6770556
Log likelihood   =  -18.9887235                   BIC             =  -209.6506

------------------------------------------------------------------------------------
                   |                 OIM
Congitivedomain2SD | Risk Ratio   Std. Err.      z    P>|z|     [95% Conf. Interval]
-------------------+----------------------------------------------------------------
          CongZIKA |   9.272727   5.786977     3.57   0.000     2.728882    31.50868
             _cons |   .0588235   .0329478    -5.06   0.000     .0196238    .1763272
------------------------------------------------------------------------------------
Note: _cons estimates baseline risk.

. glm Congitive CongZIKA Maternalinfectioninthe1sttr, fam(bin) link(log) nolog eform

Generalized linear models                         Number of obs   =         62
Optimization     : ML                             Residual df     =         59
                                                  Scale parameter =          1
Deviance         =  35.61810621                   (1/df) Deviance =   .6036967
Pearson          =  55.36963588                   (1/df) Pearson  =   .9384684

Variance function: V(u) = u*(1-u/1)               [Binomial]
Link function    : g(u) = ln(u)                   [Log]

                                                  AIC             =   .6712598
Log likelihood   =  -17.8090531                   BIC             =  -207.8828

---------------------------------------------------------------------------------------------
                            |                 OIM
         Congitivedomain2SD | Risk Ratio   Std. Err.      z    P>|z|     [95% Conf. Interval]
----------------------------+----------------------------------------------------------------
                   CongZIKA |   8.286445    5.18194     3.38   0.001     2.432586    28.22724
Maternalinfectioninthe1sttr |   2.114485   1.074745     1.47   0.141     .7808331    5.725995
                      _cons |   .0447738   .0271193    -5.13   0.000     .0136602     .146755
---------------------------------------------------------------------------------------------
Note: _cons estimates baseline risk.

.
```

```
. tab Socioaffectivedomain2SD CongZIKA, freq

Socio-affe |
     ctive |
    domain |      CongZIKA
     <-2SD |         0          1 |     Total
-----------+----------------------+----------
         0 |        45          7 |        52
         1 |         6          4 |        10
-----------+----------------------+----------
     Total |        51         11 |        62

. glm Socioaffectivedomain2SD CongZIKA, fam(bin) link(log) nolog eform

Generalized linear models                          Number of obs   =         62
Optimization     : ML                              Residual df     =         60
                                                   Scale parameter =          1
Deviance         =  51.36607585                    (1/df) Deviance =   .8561013
Pearson          =           62                    (1/df) Pearson  =   1.033333

Variance function: V(u) = u*(1-u/1)                [Binomial]
Link function    : g(u) = ln(u)                    [Log]

                                                   AIC             =   .8930012
Log likelihood   = -25.68303793                    BIC             =   -196.262

-----------------------------------------------------------------------------------------
                        |                 OIM
Socioaffectivedomain2SD | Risk Ratio   Std. Err.      z    P>|z|     [95% Conf. Interval]
------------------------+----------------------------------------------------------------
               CongZIKA |   3.090909   1.710225     2.04   0.041     1.044984    9.142455
                  _cons |   .1176471   .0451156    -5.58   0.000      .055483    .2494606
-----------------------------------------------------------------------------------------
Note: _cons estimates baseline risk.

. glm Socioaffectivedomain2SD CongZIKA Maternalinfectioninthe1sttr, fam(bin) link(log) nolog eform

Generalized linear models                          Number of obs   =         62
Optimization     : ML                              Residual df     =         59
                                                   Scale parameter =          1
Deviance         =   48.7697619                    (1/df) Deviance =   .8266061
Pearson          =  67.94200592                    (1/df) Pearson  =   1.151559

Variance function: V(u) = u*(1-u/1)                [Binomial]
Link function    : g(u) = ln(u)                    [Log]

                                                   AIC             =   .8833833
Log likelihood   = -24.38488095                    BIC             =  -194.7312

---------------------------------------------------------------------------------------------
                            |                 OIM
    Socioaffectivedomain2SD | Risk Ratio   Std. Err.      z    P>|z|     [95% Conf. Interval]
----------------------------+----------------------------------------------------------------
                   CongZIKA |    3.28655   1.698334     2.30   0.021     1.193656    9.049011
Maternalinfectioninthe1sttr |   2.424121    1.27574     1.68   0.092     .8641584    6.800101
                      _cons |   .0810377   .0388727    -5.24   0.000     .0316502    .2074902
---------------------------------------------------------------------------------------------
```

Note: _cons estimates baseline risk.

```
.
. tab Neurodevelopment2SDat3y CongZIKA if Maternalinfectioninthe1sttr==1

Neurodevel |
   opment |
  <-2SD at |      CongZIKA
       3y |        0          1 |     Total
-----------+----------------------+----------
         0 |       12          1 |        13
         1 |        3          3 |         6
-----------+----------------------+----------
     Total |       15          4 |        19

. tab Neurodevelopment2SDat3y CongZIKA if Maternalinfectioninthe1sttr==0

Neurodevel |
   opment |
  <-2SD at |      CongZIKA
       3y |        0          1 |     Total
-----------+----------------------+----------
         0 |       32          3 |        35
         1 |        4          4 |         8
-----------+----------------------+----------
     Total |       36          7 |        43

. cs Neurodevelopment2SDat3y CongZIKA, by(Maternalinfectioninthe1sttr)

Maternal infecti |       RR       [95% Conf. Interval]   M-H Weight
-----------------+-------------------------------------------------
               0 |   5.142857     1.669925   15.83842     .6511628
               1 |       3.75     1.176125   11.95663     .6315789
-----------------+-------------------------------------------------
           Crude |   4.636364     2.041238    10.5308
    M-H combined |   4.457061     1.989707   9.984078
-------------------------------------------------------------------
Test of homogeneity (M-H)      chi2(1) =    0.147  Pr>chi2 = 0.7010


.
.
. log close
      name:  <unnamed>
       log:  \\HOME\lpomar\Mes Documents\Zika_enfants-Guyane\Nature\R1\Dataset\Table2.log
  log type:  text
 closed on:  23 Mar 2021, 17:55:04
---------------------------------------------------------------------------------------------------------------------------------------------------
------------------------------------
```

```
-------------------------------------------------------------------------------------------------------------------------------------
----------------------------------
      name:  <unnamed>
       log:  \\HOME\lpomar\Mes Documents\Zika_enfants-Guyane\Nature\R1\Dataset\Tables3-4.log
  log type:  text
 opened on:  24 Mar 2021, 20:51:56

. import excel "\\HOME\lpomar\Mes Documents\Zika_enfants-Guyane\Nature\R1\Dataset\Source Data.xlsx",
sheet("Tables 3 & 4") firstrow
(44 vars, 140 obs)


.
. destring CongZIKA HCatbirthcm HCat1mcm HCat2mcm HCat1ycm HCat2ycm HCat3ycm Birthweightg
Weightat1mkg Weightat2mKg Weightat1ykg Weightat2yKg Weightat3yKg Structuralbrainanomal
> ies Corticaldevelopmentanomaly Corpuscallosumanomaly Calcificationsorcysticlesions Posteriorfossaanomaly
Ventriculomegaly Microphtalmy AbnormalOAE Neurologicalsymptomsat2m Ar
> throgryposis Hypertonia Dysphagiaswallowingdisorders Seizures Neurologicimpairmentsat2y Cerebralpalsyat2y
Severedystoniaortremors Seizuresat2y Delayinmotoracquisitions Ageats
> ittingpositionm Ageatwalkingm Neurosensoryalterations Impairedresponsetovisualstim
Impairedresponsetoauditoryst Neurodevelopment2SDat3y Motordomain2SD Congitivedomain2SD Soci
> oaffectivedomain2SD, replace
CongZIKA: all characters numeric; replaced as byte
(11 missing values generated)
HCatbirthcm already numeric; no replace
HCat1mcm: all characters numeric; replaced as double
(12 missing values generated)
HCat2mcm: all characters numeric; replaced as byte
(12 missing values generated)
HCat1ycm: all characters numeric; replaced as double
(28 missing values generated)
HCat2ycm: all characters numeric; replaced as double
(30 missing values generated)
HCat3ycm: all characters numeric; replaced as double
(78 missing values generated)
Birthweightg already numeric; no replace
Weightat1mkg: all characters numeric; replaced as double
(6 missing values generated)
Weightat2mKg: all characters numeric; replaced as double
(7 missing values generated)
Weightat1ykg: all characters numeric; replaced as double
(22 missing values generated)
Weightat2yKg: all characters numeric; replaced as double
(25 missing values generated)
Weightat3yKg: all characters numeric; replaced as double
(72 missing values generated)
Structuralbrainanomalies: all characters numeric; replaced as byte
(11 missing values generated)
Corticaldevelopmentanomaly: all characters numeric; replaced as byte
(11 missing values generated)
Corpuscallosumanomaly: all characters numeric; replaced as byte
(11 missing values generated)
Calcificationsorcysticlesions: all characters numeric; replaced as byte
(11 missing values generated)
Posteriorfossaanomaly: all characters numeric; replaced as byte
(11 missing values generated)
Ventriculomegaly: all characters numeric; replaced as byte
(11 missing values generated)
```

```
Microphtalmy: all characters numeric; replaced as byte
(11 missing values generated)
AbnormalOAE: all characters numeric; replaced as byte
(85 missing values generated)
Neurologicalsymptomsat2m: all characters numeric; replaced as byte
(11 missing values generated)
Arthrogryposis: all characters numeric; replaced as byte
(11 missing values generated)
Hypertonia: all characters numeric; replaced as byte
(11 missing values generated)
Dysphagiaswallowingdisorders: all characters numeric; replaced as byte
(11 missing values generated)
Seizures: all characters numeric; replaced as byte
(11 missing values generated)
Neurologicimpairmentsat2y: all characters numeric; replaced as byte
(29 missing values generated)
Cerebralpalsyat2y: all characters numeric; replaced as byte
(29 missing values generated)
Severedystoniaortremors: all characters numeric; replaced as byte
(29 missing values generated)
Seizuresat2y: all characters numeric; replaced as byte
(29 missing values generated)
Delayinmotoracquisitions: all characters numeric; replaced as byte
(27 missing values generated)
Ageatsittingpositionm: all characters numeric; replaced as double
(28 missing values generated)
Ageatwalkingm: all characters numeric; replaced as double
(29 missing values generated)
Neurosensoryalterations: all characters numeric; replaced as byte
(29 missing values generated)
Impairedresponsetovisualstim: all characters numeric; replaced as byte
(27 missing values generated)
Impairedresponsetoauditoryst: all characters numeric; replaced as byte
(27 missing values generated)
Neurodevelopment2SDat3y: all characters numeric; replaced as byte
(78 missing values generated)
Motordomain2SD: all characters numeric; replaced as byte
(78 missing values generated)
Congitivedomain2SD: all characters numeric; replaced as byte
(78 missing values generated)
Socioaffectivedomain2SD: all characters numeric; replaced as byte
(78 missing values generated)


.
. * Neonatal demise & NICU admission:
. tab Neonataldemise CongZIKA, exact

  Neonatal |       CongZIKA
    demise |         0          1 |     Total
-----------+----------------------+----------
         0 |       111         17 |       128 
         1 |         0          1 |         1 
-----------+----------------------+----------
     Total |       111         18 |       129 

           Fisher's exact =                 0.140
   1-sided Fisher's exact =                 0.140
```

```
. tab NICU CongZIKA, chi2

      NICU |      CongZIKA
 admission |         0          1 |     Total
-----------+----------------------+----------
         0 |        97         15 |       112 
         1 |        14          3 |        17 
-----------+----------------------+----------
     Total |       111         18 |       129 

          Pearson chi2(1) =   0.2225   Pr = 0.637

. 
. * HC:
. codebook HCatbirth if CongZIKA==1

---------------------------------------------------------------------------------------------------------------------------------------------------------------------------
HCatbirthcm                                                                                                                          HC at
birth (cm)
---------------------------------------------------------------------------------------------------------------------------------------------------------------------------

                  type:  numeric (double)

                 range:  [27.5,35.5]                  units:  .1
         unique values:  10                       missing .:  0/18

                  mean:   32.5556
              std. dev:   1.84621

           percentiles:        10%       25%       50%       75%       90%
                                31      31.5     32.75        34        35

. codebook HCatbirth if CongZIKA==0

---------------------------------------------------------------------------------------------------------------------------------------------------------------------------
HCatbirthcm                                                                                                                          HC at
birth (cm)
---------------------------------------------------------------------------------------------------------------------------------------------------------------------------

                  type:  numeric (double)

                 range:  [29,38]                      units:  .1
         unique values:  14                       missing .:  0/111

                  mean:   33.3784
              std. dev:   1.70056

           percentiles:        10%       25%       50%       75%       90%
                                31      32.5        33      34.5      35.5

. ranksum HCatbirth, by(CongZIKA)

Two-sample Wilcoxon rank-sum (Mann-Whitney) test
```

```
    CongZIKA |      obs    rank sum    expected
-------------+---------------------------------
           0 |      111        7471        7215
           1 |       18         914        1170
-------------+---------------------------------
    combined |      129        8385        8385

unadjusted variance    21645.00
adjustment for ties     -529.81
                     ----------
adjusted variance      21115.19

Ho: HCatbi~m(CongZIKA==0) = HCatbi~m(CongZIKA==1)
             z =   1.762
    Prob > |z| =   0.0781
    Exact Prob =   0.0784

.
. codebook HCat1m if CongZIKA==1

--------------------------------------------------------------------------------------------------------------------------------------------------------------------------
HCat1mcm                                                                                                                                                      HC at
1m (cm)
--------------------------------------------------------------------------------------------------------------------------------------------------------------------------

                  type:  numeric (double)

                 range:  [31,39]                      units:  1
         unique values:  8                        missing .:  1/18

            tabulation:  Freq.  Value
                             1  31
                             2  33
                             1  34
                             1  35
                             3  36
                             4  37
                             4  38
                             1  39
                             1  .

. codebook HCat1m if CongZIKA==0

--------------------------------------------------------------------------------------------------------------------------------------------------------------------------
HCat1mcm                                                                                                                                                      HC at
1m (cm)
--------------------------------------------------------------------------------------------------------------------------------------------------------------------------

                  type:  numeric (double)

                 range:  [33,40]                      units:  .1
         unique values:  11                       missing .:  0/111
```

```
                 mean:   36.536
             std. dev:   1.60922

          percentiles:        10%       25%       50%       75%       90%
                               34        36        37        38        39

. ranksum HCat1m, by(CongZIKA)

Two-sample Wilcoxon rank-sum (Mann-Whitney) test

   CongZIKA |      obs    rank sum    expected
-------------+---------------------------------
          0 |      111        7226      7159.5
          1 |       17        1030      1096.5
-------------+---------------------------------
   combined |      128        8256        8256

unadjusted variance    20285.25
adjustment for ties     -639.72
                     ----------
adjusted variance      19645.53

Ho: HCat1mcm(CongZIKA==0) = HCat1mcm(CongZIKA==1)
             z =   0.474
    Prob > |z| =   0.6352
    Exact Prob =   0.6391

.
. codebook HCat1ycm if CongZIKA==1

------------------------------------------------------------------------------------------------------------------------------------------
----------------------------------
HCat1ycm                                                                                                                        HC at 1y
(cm)
------------------------------------------------------------------------------------------------------------------------------------------
----------------------------------

                  type:  numeric (double)

                 range:  [37,47]                      units:  1
         unique values:  8                        missing .:  3/18

            tabulation:  Freq.  Value
                             1  37
                             1  41
                             1  42
                             4  43
                             4  44
                             2  45
                             1  46
                             1  47
                             3  .

. codebook HCat1ycm if CongZIKA==0

------------------------------------------------------------------------------------------------------------------------------------------
----------------------------------
```

```
HCat1ycm                                                                                                                    HC at 1y
(cm)
-----------------------------------------------------------------------------------------------------------------------------
-------------------------------

                  type:  numeric (double)

                 range:  [41,50]                      units:  .1
         unique values:  11                       missing .:  14/111

                  mean:   45.5722
              std. dev:   2.19729

           percentiles:        10%       25%       50%       75%       90%
                                42        44        46        47        48

. ranksum HCat1ycm, by(CongZIKA)

Two-sample Wilcoxon rank-sum (Mann-Whitney) test

    CongZIKA |      obs    rank sum    expected
-------------+---------------------------------
           0 |       97      5851.5      5480.5
           1 |       15       476.5       847.5
-------------+---------------------------------
    combined |      112        6328        6328

unadjusted variance    13701.25
adjustment for ties     -244.67
                     ----------
adjusted variance      13456.58

Ho: HCat1ycm(CongZIKA==0) = HCat1ycm(CongZIKA==1)
             z =   3.198
    Prob > |z| =   0.0014
    Exact Prob =   0.0010


.
. codebook HCat2ycm if CongZIKA==1

-----------------------------------------------------------------------------------------------------------------------------
-------------------------------
HCat2ycm                                                                                                                    HC at 2y
(cm)
-----------------------------------------------------------------------------------------------------------------------------
-------------------------------

                  type:  numeric (double)

                 range:  [38,50]                      units:  1
         unique values:  8                        missing .:  3/18

            tabulation:  Freq.  Value
                             1  38
                             1  44
                             2  45
                             4  46
```

```
                    1  47
                    3  48
                    2  49
                    1  50
                    3  .

. codebook HCat2ycm if CongZIKA==0

--------------------------------------------------------------------------------------------------------------
-------------------------------
HCat2ycm                                                                                            HC at 2y
(cm)
--------------------------------------------------------------------------------------------------------------
-------------------------------

                  type:  numeric (double)

                 range:  [44,53]                      units:  .1
         unique values:  11                       missing .:  16/111

                  mean:   48.4053
              std. dev:   2.14537

           percentiles:        10%       25%       50%       75%       90%
                                45        47        48        50        51

. ranksum HCat2ycm, by(CongZIKA)

Two-sample Wilcoxon rank-sum (Mann-Whitney) test

    CongZIKA |      obs    rank sum    expected
-------------+---------------------------------
           0 |       95        5585      5272.5
           1 |       15         520       832.5
-------------+---------------------------------
    combined |      110        6105        6105

unadjusted variance    13181.25
adjustment for ties     -242.39
                     ----------
adjusted variance      12938.86

Ho: HCat2ycm(CongZIKA==0) = HCat2ycm(CongZIKA==1)
             z =   2.747
    Prob > |z| =   0.0060
    Exact Prob =   0.0053

.
. codebook HCat3ycm if CongZIKA==1

--------------------------------------------------------------------------------------------------------------
-------------------------------
HCat3ycm                                                                                            HC at 3y
(cm)
--------------------------------------------------------------------------------------------------------------
-------------------------------
```

```
                 type:  numeric (double)

                range:  [39,53]                      units:  1
        unique values:  5                        missing .:  7/18

           tabulation:  Freq.  Value
                            1  39
                            2  49
                            4  50
                            3  51
                            1  53
                            7  .

. codebook HCat3ycm if CongZIKA==0

------------------------------------------------------------------------------------------------------------------------------------
----------------------------------
HCat3ycm                                                                                                                   HC at 3y
(cm)
------------------------------------------------------------------------------------------------------------------------------------
----------------------------------

                 type:  numeric (double)

                range:  [45,54]                      units:  .1
        unique values:  12                       missing .:  60/111

                 mean:   50.5784
             std. dev:   1.80381

          percentiles:        10%       25%       50%       75%       90%
                               48        50      50.5        52        53

. ranksum HCat3ycm, by(CongZIKA)

Two-sample Wilcoxon rank-sum (Mann-Whitney) test

    CongZIKA |      obs    rank sum    expected
-------------+---------------------------------
           0 |       51      1660.5      1606.5
           1 |       11       292.5       346.5
-------------+---------------------------------
    combined |       62        1953        1953

unadjusted variance     2945.25
adjustment for ties     -103.09
                      ----------
adjusted variance       2842.16

Ho: HCat3ycm(CongZIKA==0) = HCat3ycm(CongZIKA==1)
             z =   1.013
    Prob > |z| =   0.3111
    Exact Prob =   0.3189

.
. * Weight:
. codebook Birthweightg if CongZIKA==1
```

```
--------------------------------------------------------------------------------------------------------------------------------------
----------------------------------
Birthweightg
Birthweight (g)
--------------------------------------------------------------------------------------------------------------------------------------
----------------------------------

                type:  numeric (double)

               range:  [1910,3310]                units:  1
       unique values:  17                     missing .:  0/18

                mean:   2791.61
            std. dev:    353.75

         percentiles:        10%       25%       50%       75%       90%
                            2160      2675      2905      2970      3190

. codebook Birthweightg if CongZIKA==0

--------------------------------------------------------------------------------------------------------------------------------------
----------------------------------
Birthweightg
Birthweight (g)
--------------------------------------------------------------------------------------------------------------------------------------
----------------------------------

                type:  numeric (double)

               range:  [1930,4620]                units:  1
       unique values:  98                     missing .:  0/111

                mean:   3144.13
            std. dev:   490.859

         percentiles:        10%       25%       50%       75%       90%
                            2455      2755      3185      3495      3665

. ranksum Birthweightg, by(CongZIKA)

Two-sample Wilcoxon rank-sum (Mann-Whitney) test

   CongZIKA |      obs    rank sum    expected
-------------+---------------------------------
          0 |      111      7649.5        7215
          1 |       18       735.5        1170
-------------+---------------------------------
   combined |      129        8385        8385

unadjusted variance    21645.00
adjustment for ties       -1.15
                     ----------
adjusted variance      21643.85

Ho: Birthw~g(CongZIKA==0) = Birthw~g(CongZIKA==1)
             z =   2.953
    Prob > |z| =   0.0031
```

```
    Exact Prob =   0.0027

.
. codebook Weightat1mkg if CongZIKA==1

-----------------------------------------------------------------------------------------------------------------------------------
-----------------------------------
Weightat1mkg                                                                                                           Weight at
1m (kg)
-----------------------------------------------------------------------------------------------------------------------------------
-----------------------------------

                  type:  numeric (double)

                 range:  [3,4.1]                      units:  .1
         unique values:  9                          missing .:  1/18

            tabulation:  Freq.  Value
                             1  3
                             1  3.2
                             1  3.4
                             2  3.5
                             3  3.7
                             5  3.8
                             2  3.9
                             1  4
                             1  4.1
                             1  .

. codebook Weightat1mkg if CongZIKA==0

-----------------------------------------------------------------------------------------------------------------------------------
-----------------------------------
Weightat1mkg                                                                                                           Weight at
1m (kg)
-----------------------------------------------------------------------------------------------------------------------------------
-----------------------------------

                  type:  numeric (double)

                 range:  [2.4,5.4]                    units:  .01
         unique values:  28                         missing .:  0/111

                  mean:   4.05315
              std. dev:   .549577

           percentiles:        10%       25%       50%       75%       90%
                               3.4       3.7       4.2       4.4       4.7

. ranksum Weightat1mkg, by(CongZIKA)

Two-sample Wilcoxon rank-sum (Mann-Whitney) test

    CongZIKA |      obs    rank sum    expected
-------------+---------------------------------
           0 |      111        7613      7159.5
           1 |       17         643      1096.5
```

```
-------------+--------------------------------
    combined |      128        8256        8256

unadjusted variance    20285.25
adjustment for ties      -95.07
                     ----------
adjusted variance      20190.18

Ho: Weig~mkg(CongZIKA==0) = Weig~mkg(CongZIKA==1)
             z =   3.192
    Prob > |z| =   0.0014
    Exact Prob =   0.0011

.
. codebook Weightat2m if CongZIKA==1

--------------------------------------------------------------------------------------------------------------------------------------------
-----------------------------------
Weightat2mKg                                                                                                                     Weight at
2m (Kg)
--------------------------------------------------------------------------------------------------------------------------------------------
-----------------------------------

                  type:  numeric (double)

                 range:  [4,6.2]                      units:  .1
         unique values:  10                       missing .:  1/18

                  mean:   4.85294
              std. dev:   .458418

           percentiles:        10%       25%       50%       75%       90%
                               4.4       4.6       4.8         5       5.3

. codebook Weightat2m if CongZIKA==0

--------------------------------------------------------------------------------------------------------------------------------------------
-----------------------------------
Weightat2mKg                                                                                                                     Weight at
2m (Kg)
--------------------------------------------------------------------------------------------------------------------------------------------
-----------------------------------

                  type:  numeric (double)

                 range:  [3.5,6.5]                    units:  .1
         unique values:  25                       missing .:  1/111

                  mean:   5.18455
              std. dev:   .670641

           percentiles:        10%       25%       50%       75%       90%
                               4.2       4.7       5.2       5.7         6

. ranksum Weightat2m, by(CongZIKA)

Two-sample Wilcoxon rank-sum (Mann-Whitney) test
```

```
   CongZIKA |      obs    rank sum    expected
-------------+---------------------------------
          0 |      110      7362.5        7040
          1 |       17       765.5        1088
-------------+---------------------------------
   combined |      127        8128        8128

unadjusted variance    19946.67
adjustment for ties      -58.55
                     ----------
adjusted variance      19888.12

Ho: Weig~mKg(CongZIKA==0) = Weig~mKg(CongZIKA==1)
             z =   2.287
    Prob > |z| =   0.0222
    Exact Prob =   0.0214

.
. codebook Weightat1y if CongZIKA==1

-----------------------------------------------------------------------------------------------------------------------------
------------------------------------
Weightat1ykg                                                                                                       Weight at
1y (kg)
-----------------------------------------------------------------------------------------------------------------------------
------------------------------------

                  type:  numeric (double)

                 range:  [6,11.3]                     units:  .1
         unique values:  13                       missing .:  3/18

                  mean:   9.63333
              std. dev:   1.37616

           percentiles:        10%       25%       50%       75%       90%
                                 8       8.9        10      10.7      11.2

. codebook Weightat1y if CongZIKA==0

-----------------------------------------------------------------------------------------------------------------------------
------------------------------------
Weightat1ykg                                                                                                       Weight at
1y (kg)
-----------------------------------------------------------------------------------------------------------------------------
------------------------------------

                  type:  numeric (double)

                 range:  [6.4,13.3]                   units:  .1
         unique values:  45                       missing .:  14/111

                  mean:   10.0299
              std. dev:   1.34507

           percentiles:        10%       25%       50%       75%       90%
                               8.3       8.9      10.1      10.9      11.8
```

```
. ranksum Weightat1y, by(CongZIKA)

Two-sample Wilcoxon rank-sum (Mann-Whitney) test

    CongZIKA |      obs    rank sum    expected
-------------+---------------------------------
           0 |       97        5558      5480.5
           1 |       15         770       847.5
-------------+---------------------------------
    combined |      112        6328        6328

unadjusted variance    13701.25
adjustment for ties      -12.00
                     ----------
adjusted variance      13689.25

Ho: Weig~ykg(CongZIKA==0) = Weig~ykg(CongZIKA==1)
             z =   0.662
    Prob > |z| =   0.5077
    Exact Prob =   0.5128

.
. codebook Weightat2y if CongZIKA==1

---------------------------------------------------------------------------------------------------------------------------------------------------------------------------------
Weightat2yKg                                                                                                                                                         Weight at
2y (Kg)
---------------------------------------------------------------------------------------------------------------------------------------------------------------------------------

                  type:  numeric (double)

                 range:  [9.6,13.9]                   units:  .1
         unique values:  15                       missing .:  3/18

                  mean:   12.1333
              std. dev:   1.25338

           percentiles:        10%       25%       50%       75%       90%
                               9.8      11.5      12.3      13.1      13.8

. codebook Weightat2y if CongZIKA==0

---------------------------------------------------------------------------------------------------------------------------------------------------------------------------------
Weightat2yKg                                                                                                                                                         Weight at
2y (Kg)
---------------------------------------------------------------------------------------------------------------------------------------------------------------------------------

                  type:  numeric (double)

                 range:  [9.4,15]                     units:  .1
         unique values:  42                       missing .:  17/111

                  mean:   12.3851
```

```
           std. dev:   1.22246

         percentiles:        10%       25%       50%       75%       90%
                            10.8      11.6      12.4      13.2        14

. ranksum Weightat2y, by(CongZIKA)

Two-sample Wilcoxon rank-sum (Mann-Whitney) test

   CongZIKA |      obs    rank sum    expected
-------------+---------------------------------
          0 |       94      5223.5        5170
          1 |       15       771.5         825
-------------+---------------------------------
   combined |      109        5995        5995

unadjusted variance    12925.00
adjustment for ties      -14.61
                     ----------
adjusted variance      12910.39

Ho: Wei~2yKg(CongZIKA==0) = Wei~2yKg(CongZIKA==1)
             z =   0.471
    Prob > |z| =   0.6377
    Exact Prob =   0.6425

.
. codebook Weightat3y if CongZIKA==1

---------------------------------------------------------------------------------------------------------------------------------------------------------------
Weightat3yKg                                                                                                                                          Weight at
3y (Kg)
---------------------------------------------------------------------------------------------------------------------------------------------------------------

                type:  numeric (double)

               range:  [11.4,20]                    units:  .1
       unique values:  11                       missing .:  6/18

                mean:     15.15
            std. dev:   2.15259

         percentiles:        10%       25%       50%       75%       90%
                            13.9      14.2      14.6     16.05      17.5

. codebook Weightat3y if CongZIKA==0

---------------------------------------------------------------------------------------------------------------------------------------------------------------
Weightat3yKg                                                                                                                                          Weight at
3y (Kg)
---------------------------------------------------------------------------------------------------------------------------------------------------------------

                type:  numeric (double)
```

```
              range:  [12,19.4]                  units:  .1
        unique values:  35                      missing .:  61/111

               mean:   14.826
            std. dev:   1.54521

          percentiles:        10%       25%       50%       75%       90%
                              12.9      13.8      14.7      15.8     16.85

. ranksum Weightat3y, by(CongZIKA)

Two-sample Wilcoxon rank-sum (Mann-Whitney) test

    CongZIKA |      obs    rank sum    expected
-------------+---------------------------------
           0 |       50      1547.5        1575
           1 |       12       405.5         378
-------------+---------------------------------
    combined |       62        1953        1953

unadjusted variance     3150.00
adjustment for ties       -4.20
                      ----------
adjusted variance       3145.80

Ho: Wei~3yKg(CongZIKA==0) = Wei~3yKg(CongZIKA==1)
             z =  -0.490
    Prob > |z| =   0.6239
    Exact Prob =   0.6313


.
. * Structural brain anomalies:
. tab Structuralbrainanomalies CongZIKA, chi2

Structural |
     brain |       CongZIKA
 anomalies |         0          1 |     Total
-----------+----------------------+----------
         0 |       108         12 |       120
         1 |         3          6 |         9
-----------+----------------------+----------
     Total |       111         18 |       129

          Pearson chi2(1) =  22.3910   Pr = 0.000

. tab Cortical CongZIKA, exact

  Cortical |
developmen |       CongZIKA
t anomaly  |         0          1 |     Total
-----------+----------------------+----------
         0 |       111         14 |       125
         1 |         0          4 |         4
-----------+----------------------+----------
     Total |       111         18 |       129

           Fisher's exact =                 0.000
```

```
  1-sided Fisher's exact =                 0.000

. tab Corpuscall CongZIKA, exact

    Corpus |
  callosum |       CongZIKA
   anomaly |         0          1 |     Total
-----------+----------------------+----------
         0 |       109         14 |       123
         1 |         2          4 |         6
-----------+----------------------+----------
     Total |       111         18 |       129

           Fisher's exact =                 0.003
   1-sided Fisher's exact =                 0.003

. tab Calcifications CongZIKA, exact

Calcificat |
   ions or |
    cystic |       CongZIKA
   lesions |         0          1 |     Total
-----------+----------------------+----------
         0 |       110         13 |       123
         1 |         1          5 |         6
-----------+----------------------+----------
     Total |       111         18 |       129

           Fisher's exact =                 0.000
   1-sided Fisher's exact =                 0.000

. tab Posteriorfossa CongZIKA, exact

 Posterior |
     fossa |       CongZIKA
   anomaly |         0          1 |     Total
-----------+----------------------+----------
         0 |       111         14 |       125
         1 |         0          4 |         4
-----------+----------------------+----------
     Total |       111         18 |       129

           Fisher's exact =                 0.000
   1-sided Fisher's exact =                 0.000

. tab Ventriculomegaly CongZIKA, exact

Ventriculo |       CongZIKA
   megaly  |         0          1 |     Total
-----------+----------------------+----------
         0 |       110         14 |       124
         1 |         1          4 |         5
-----------+----------------------+----------
     Total |       111         18 |       129

           Fisher's exact =                 0.001
   1-sided Fisher's exact =                 0.001
```

```
.
. * Ocular anomalies
. tab Microphtalmy CongZIKA, exact

Microphtal |      CongZIKA
       my |         0          1 |     Total
-----------+----------------------+----------
         0 |       111         17 |       128 
         1 |         0          1 |         1 
-----------+----------------------+----------
     Total |       111         18 |       129 

           Fisher's exact =                 0.140
   1-sided Fisher's exact =                 0.140

. tab Fundoscopy CongZIKA, exact

Enumerating sample-space combinations:
stage 5:  enumerations = 1
stage 4:  enumerations = 2
stage 3:  enumerations = 5
stage 2:  enumerations = 7
stage 1:  enumerations = 0

                      |      CongZIKA
           Fundoscopy |         0          1 |     Total
----------------------+----------------------+----------
                    . |        75          9 |        84 
                    0 |        34          6 |        40 
Macula atrophy, cho.. |         1          1 |         2 
Subretinal hemorrhage |         1          1 |         2 
Subretinal hemorrha.. |         0          1 |         1 
----------------------+----------------------+----------
                Total |       111         18 |       129 

           Fisher's exact =                 0.033

. cci 3 2 8 32
                                                         Proportion
                 |   Exposed   Unexposed  |      Total     Exposed
-----------------+------------------------+------------------------
           Cases |         3           2  |          5       0.6000
        Controls |         8          32  |         40       0.2000
-----------------+------------------------+------------------------
           Total |        11          34  |         45       0.2444
                 |                        |
                 |      Point estimate    |    [95% Conf. Interval]
                 |------------------------+------------------------
      Odds ratio |                 6      |   .5561587    79.06791 (exact)
 Attr. frac. ex. |          .8333333      |  -.7980481    .9873526 (exact)
 Attr. frac. pop |                .5      |
                 +-------------------------------------------------
                               chi2(1) =     3.85  Pr>chi2 = 0.0497

. cci 2 1 8 33
                                                         Proportion
                 |   Exposed   Unexposed  |      Total     Exposed
```

```
-----------------+------------------------+------------------------
           Cases |        2           1  |          3       0.6667
        Controls |        8          33  |         41       0.1951
-----------------+------------------------+------------------------
           Total |       10          34  |         44       0.2273
                 |                        |
                 |      Point estimate    |    [95% Conf. Interval]
                 |------------------------+------------------------
      Odds ratio |             8.25       |    .3613468     500.877 (exact)
 Attr. frac. ex. |         .8787879       |   -1.767424    .9980035 (exact)
 Attr. frac. pop |         .5858586       |
                 +-------------------------------------------------
                               chi2(1) =     3.54  Pr>chi2 = 0.0599

. cci 1 1 9 33
                                                         Proportion
                 |   Exposed   Unexposed  |      Total     Exposed
-----------------+------------------------+------------------------
           Cases |        1           1  |          2       0.5000
        Controls |        9          33  |         42       0.2143
-----------------+------------------------+------------------------
           Total |       10          34  |         44       0.2273
                 |                        |
                 |      Point estimate    |    [95% Conf. Interval]
                 |------------------------+------------------------
      Odds ratio |         3.666667       |    .0420734    296.3017 (exact)
 Attr. frac. ex. |         .7272727       |   -22.76798    .9966251 (exact)
 Attr. frac. pop |         .3636364       |
                 +-------------------------------------------------
                               chi2(1) =     0.89  Pr>chi2 = 0.3462

. 
. * OAE
. tab AbnormalOAE CongZIKA, exact

  Abnormal |       CongZIKA
       OAE |         0          1 |     Total
-----------+----------------------+----------
         0 |        42         10 |        52 
         1 |         1          2 |         3 
-----------+----------------------+----------
     Total |        43         12 |        55 

           Fisher's exact =                 0.117
   1-sided Fisher's exact =                 0.117

. 
. * Severe neurological symptoms:
. tab Neurologicalsymptomsat2m CongZIKA, exact

Neurologic |
        al |
  symptoms |       CongZIKA
    at 2m |         0          1 |     Total
-----------+----------------------+----------
         0 |       110         13 |       123 
         1 |         1          5 |         6 
```

```
-----------+----------------------+----------
     Total |       111         18 |       129

          Fisher's exact =                 0.000
  1-sided Fisher's exact =                 0.000

. tab Arthrogryposis CongZIKA, exact

Arthrogryp |       CongZIKA
      osis |         0          1 |     Total
-----------+----------------------+----------
         0 |       111         17 |       128
         1 |         0          1 |         1
-----------+----------------------+----------
     Total |       111         18 |       129

          Fisher's exact =                 0.140
  1-sided Fisher's exact =                 0.140

. tab Hypertonia CongZIKA, exact

           |       CongZIKA
Hypertonia |         0          1 |     Total
-----------+----------------------+----------
         0 |       111         15 |       126
         1 |         0          3 |         3
-----------+----------------------+----------
     Total |       111         18 |       129

          Fisher's exact =                 0.002
  1-sided Fisher's exact =                 0.002

. tab Dysphagiaswallowingdisorders CongZIKA, exact

 Dysphagia |
         / |
swallowing |       CongZIKA
 disorders |         0          1 |     Total
-----------+----------------------+----------
         0 |       111         16 |       127
         1 |         0          2 |         2
-----------+----------------------+----------
     Total |       111         18 |       129

          Fisher's exact =                 0.019
  1-sided Fisher's exact =                 0.019

. tab Seizures CongZIKA, exact

           |       CongZIKA
  Seizures |         0          1 |     Total
-----------+----------------------+----------
         0 |       110         17 |       127
         1 |         1          1 |         2
-----------+----------------------+----------
     Total |       111         18 |       129
```

```
         Fisher's exact =                 0.261
   1-sided Fisher's exact =                 0.261


.
. tab Calcifications CongZIKA, exact

Calcificat |
   ions or |
    cystic |       CongZIKA
   lesions |         0          1 |     Total
-----------+----------------------+----------
         0 |       110         13 |       123 
         1 |         1          5 |         6 
-----------+----------------------+----------
     Total |       111         18 |       129 

         Fisher's exact =                 0.000
   1-sided Fisher's exact =                 0.000

. tab Posteriorfossa CongZIKA, exact

 Posterior |
     fossa |       CongZIKA
   anomaly |         0          1 |     Total
-----------+----------------------+----------
         0 |       111         14 |       125 
         1 |         0          4 |         4 
-----------+----------------------+----------
     Total |       111         18 |       129 

         Fisher's exact =                 0.000
   1-sided Fisher's exact =                 0.000

. tab Ventriculomegaly CongZIKA, exact

Ventriculo |       CongZIKA
   megaly  |         0          1 |     Total
-----------+----------------------+----------
         0 |       110         14 |       124 
         1 |         1          4 |         5 
-----------+----------------------+----------
     Total |       111         18 |       129 

         Fisher's exact =                 0.001
   1-sided Fisher's exact =                 0.001


.
. * Neurologicimpairmentsat 2years
. tab Neurologicimpairmentsat2y CongZIKA, chi2

Neurologic |
impairment |       CongZIKA
   s at 2y |         0          1 |     Total
-----------+----------------------+----------
         0 |        92         10 |       102 
         1 |         4          5 |         9 
-----------+----------------------+----------
```

```
    Total |        96         15 |       111 

          Pearson chi2(1) =  14.8121   Pr = 0.000

. tab Cerebralpalsyat2y CongZIKA, exact

  Cerebral |
  palsy at |       CongZIKA
        2y |         0          1 |     Total
-----------+----------------------+----------
         0 |        96         13 |       109 
         1 |         0          2 |         2 
-----------+----------------------+----------
    Total |        96         15 |       111 

           Fisher's exact =                 0.017
   1-sided Fisher's exact =                 0.017

. tab Severedystoniaortremors CongZIKA, chi2

    Severe |
  dystonia |
or tremors |       CongZIKA
     at 2y |         0          1 |     Total
-----------+----------------------+----------
         0 |        93         12 |       105 
         1 |         3          3 |         6 
-----------+----------------------+----------
    Total |        96         15 |       111 

          Pearson chi2(1) =   7.2249   Pr = 0.007

. tab Seizuresat2y CongZIKA, exact

  Seizures |       CongZIKA
     at 2y |         0          1 |     Total
-----------+----------------------+----------
         0 |        94         12 |       106 
         1 |         2          3 |         5 
-----------+----------------------+----------
    Total |        96         15 |       111 

           Fisher's exact =                 0.017
   1-sided Fisher's exact =                 0.017

. 
. * Motor acquisitions
. codebook Ageatsittingposition if CongZIKA==1

------------------------------------------------------------------------------------------------------------------------------------------------------------------------
Ageatsittingpositionm                                                                                                                      Age at sitting
position (m)
------------------------------------------------------------------------------------------------------------------------------------------------------------------------

                  type:  numeric (double)
```

```
            range:  [0,24]                      units:  .1
     unique values:  9                      missing .:  3/18

        tabulation:  Freq.  Value
                         1  0
                         1  3
                         3  5
                         1  5.5
                         4  6
                         1  7
                         2  8
                         1  14
                         1  24
                         3  .

. codebook Ageatsittingposition if CongZIKA==0

----------------------------------------------------------------------------------------------------------------------------------------------------------------------------------
Ageatsittingpositionm                                                                                              Age at sitting
position (m)
----------------------------------------------------------------------------------------------------------------------------------------------------------------------------------


             type:  numeric (double)

            range:  [4,11]                      units:  .1
     unique values:  10                     missing .:  14/111

             mean:   6.22165
         std. dev:   1.44692

      percentiles:        10%       25%       50%       75%       90%
                            4         5         6         7         8

. ranksum Ageatsittingposition, by(CongZIKA)

Two-sample Wilcoxon rank-sum (Mann-Whitney) test

    CongZIKA |      obs    rank sum    expected
-------------+---------------------------------
           0 |       97        5508      5480.5
           1 |       15         820       847.5
-------------+---------------------------------
    combined |      112        6328        6328

unadjusted variance    13701.25
adjustment for ties     -671.50
                     ----------
adjusted variance      13029.75

Ho: Ageats~m(CongZIKA==0) = Ageats~m(CongZIKA==1)
             z =   0.241
    Prob > |z| =   0.8096
    Exact Prob =   0.8113

. gen delaysit=0
```

```
. replace delaysit=1 if (Ageatsittingposition>9 and Ageatsittingposition!=.)
9andAgeatsittingposition invalid name
r(198);

end of do-file

r(198);

. do "C:\Users\lpomar\AppData\Local\Temp\STD1d6c_000000.tmp"

. replace delaysit=1 if (Ageatsittingposition>9 & Ageatsittingposition!=.)
(3 real changes made)

. tab delaysit CongZIKA, exact

           |       CongZIKA
  delaysit |         0          1 |     Total
-----------+----------------------+----------
         0 |       110         16 |       126 
         1 |         1          2 |         3 
-----------+----------------------+----------
     Total |       111         18 |       129 

           Fisher's exact =                 0.051
   1-sided Fisher's exact =                 0.051

.
. codebook Ageatwalkingm if CongZIKA==1

----------------------------------------------------------------------------------------------------------------------------------
------------------------------------
Ageatwalkingm                                                                                                          Age at
walking (m)
----------------------------------------------------------------------------------------------------------------------------------
------------------------------------

                  type:  numeric (double)

                 range:  [0,24]                       units:  .1
         unique values:  9                        missing .:  3/18

            tabulation:  Freq.  Value
                             1  0
                             2  8
                             1  9
                             2  10
                             4  11
                             2  12
                             1  12.5
                             1  18
                             1  24
                             3  .

. codebook Ageatwalkingm if CongZIKA==0

----------------------------------------------------------------------------------------------------------------------------------
------------------------------------
```

```
Ageatwalkingm                                                                          Age at
walking (m)
----------------------------------------------------------------------------------------------------------------------------
--------------------------------

                  type:  numeric (double)

                 range:  [7,17]                       units:  1
         unique values:  11                       missing .:  15/111

                  mean:   11.4271
             std. dev:   2.03001

           percentiles:        10%       25%       50%       75%       90%
                               9        10        11        12        14

. ranksum Ageatwalkingm, by(CongZIKA)

Two-sample Wilcoxon rank-sum (Mann-Whitney) test

    CongZIKA |      obs    rank sum    expected
-------------+---------------------------------
           0 |       96      5488.5        5376
           1 |       15       727.5         840
-------------+---------------------------------
    combined |      111        6216        6216

unadjusted variance    13440.00
adjustment for ties     -426.93
                     ----------
adjusted variance      13013.07

Ho: Ageatw~m(CongZIKA==0) = Ageatw~m(CongZIKA==1)
             z =   0.986
    Prob > |z| =   0.3240
    Exact Prob =   0.3289

. gen delaywalk=0

. replace delaywalk=1 if (Ageatwalking>18 & Ageatwalking!=.)
(1 real change made)

. tab delaywalk CongZIKA, exact

           |       CongZIKA
 delaywalk |         0          1 |     Total
-----------+----------------------+----------
         0 |       111         17 |       128
         1 |         0          1 |         1
-----------+----------------------+----------
     Total |       111         18 |       129

           Fisher's exact =                 0.140
   1-sided Fisher's exact =                 0.140

.
. * Vision and hearing evaluation:
```

```
. tab Impairedresponsetovisualstim CongZIKA, chi2

  Impaired |
  response |
 to visual |       CongZIKA
   stimuli |         0          1 |     Total
-----------+----------------------+----------
         0 |        97         13 |       110
         1 |         1          2 |         3
-----------+----------------------+----------
     Total |        98         15 |       113

          Pearson chi2(1) =   7.6314   Pr = 0.006

. tab Impairedresponsetoauditoryst CongZIKA, chi2

  Impaired |
  response |
        to |
  auditory |       CongZIKA
   stimuli |         0          1 |     Total
-----------+----------------------+----------
         0 |        97         13 |       110
         1 |         1          2 |         3
-----------+----------------------+----------
     Total |        98         15 |       113

          Pearson chi2(1) =   7.6314   Pr = 0.006


.
. * CDAS
. tab Neurodevelopment2SDat3y CongZIKA, chi2

Neurodevel |
    opment |
  <-2SD at |       CongZIKA
        3y |         0          1 |     Total
-----------+----------------------+----------
         0 |        44          4 |        48
         1 |         7          7 |        14
-----------+----------------------+----------
     Total |        51         11 |        62

          Pearson chi2(1) =  12.8936   Pr = 0.000

. tab Motordomain2SD CongZIKA, exact

     Motor |
    domain |       CongZIKA
     <-2SD |         0          1 |     Total
-----------+----------------------+----------
         0 |        50          9 |        59
         1 |         1          2 |         3
-----------+----------------------+----------
     Total |        51         11 |        62

           Fisher's exact =                 0.079
```

```
   1-sided Fisher's exact =                 0.079

. tab Congitivedomain2SD CongZIKA, chi2

 Congitive |
    domain |       CongZIKA
     <-2SD |         0          1 |     Total
-----------+----------------------+----------
         0 |        48          5 |        53
         1 |         3          6 |         9
-----------+----------------------+----------
     Total |        51         11 |        62

          Pearson chi2(1) =  17.2677   Pr = 0.000

. tab Socioaffectivedomain2SD CongZIKA, chi2

Socio-affe |
     ctive |
    domain |       CongZIKA
     <-2SD |         0          1 |     Total
-----------+----------------------+----------
         0 |        45          7 |        52
         1 |         6          4 |        10
-----------+----------------------+----------
     Total |        51         11 |        62

          Pearson chi2(1) =   4.0475   Pr = 0.044

.
. log close
      name:  <unnamed>
       log:  \\HOME\lpomar\Mes Documents\Zika_enfants-Guyane\Nature\R1\Dataset\Tables3-4.log
  log type:  text
 closed on:  24 Mar 2021, 20:52:26
------------------------------------------------------------------------------------------------------------------------------------------
------------------------------------
```
