# Supplementary material for: Association between confirmed congenital Zika infection at birth and outcomes up to 3 years of life
Source: Nat Commun. 2021 Jun 1;12:3270. doi: 10.1038/s41467-021-23468-3 (PMC8169933; doi:10.1038/s41467-021-23468-3)
Supplement: Supplementary file 2 — Supplementary Information [file 41467_2021_23468_MOESM2_ESM.pdf]

Supplementary Information

1) Supplementary Table 1: Details of laboratory testing for congenital Zika infection among 132 newborns from Zika-infected mothers

2) Supplementary Table 2: Baseline characteristics of infants followed up to 2years of life and children followed up to 3 years of life

3) Supplementary Table 3: Effect of maternal Zika infection diagnosed in the first trimester on main outcomes

Supplementary Table 1: Details of laboratory testing for congenital Zika infection among 132 newborns from Zika-infected mothers

	Positive	Negative	Not tested
Total	18	111	3
RT-PCR			
cord blood	1	100	31
urine	1	111	20
amniotic fluid	2	2	128
cerebrospinal fluid	1	2	129
placenta	9	103	20
IgM			
neonatal serum	12	117	3
cerebrospinal fluid	1	0	131

Supplementary Table 2: Baseline characteristics of infants followed up to 2years of life and children followed up to 3 years of life

Age, weight and term variables are presented as a median with extreme values. For qualitative variables, absolute frequencies and relative frequencies (percentages) are presented.

	Characteristics of children followed up to 2years of life (n=111)		Characteristics of children followed up to 3years of life (n=62)	
	Confirmed congenital infection at birth N= 15	Negative testing at birth N= 96	Confirmed congenital infection at birth N= 11	Negative testing at birth N= 51
Maternal age at birth (years) - median (min-max)	25 (19-38)	26 (18-43)	25 (19-38)	26 (18-38)
Maternal socio-economic status – no. (%)				
Low	4 (26.7%)	33 (34.4%)	3 (27.3%)	23 (45.1%)
Moderate	8 (43.3%)	40 (41.7%)	7 (63.6%)	21 (41.2%)
High	1 (6.7%)	7 (7.3%)	1 (9.1%)	5 (9.8%)
Unknown	2 (13.3%)	16 (16.7%)	0 (0.0%)	2 (3.9%)
Maternal exposure during pregnancy – no. (%)				
Alcohol consumption	1 (6.7%)	12 (12.5%)	1 (9.1%)	12 (23.5%)
Drug use	0 (0.0%)	1 (1.0%)	0 (0.0%)	1 (2.0%)
Current smoker	1 (6.7%)	3 (3.2%)	0 (0.0%)	2 (4.0%)
Lead poisoning	1 (6.7%)	5 (5.2%)	1 (9.1%)	3 (5.9%)
Any maternal comorbidities$^{\pi}$ – no (%)	3 (20.0%)	13 (13.5%)	3 (27.3%)	8 (15.7%)
Diabetes (previous or gestational)	1 (6.7%)	6 (6.3%)	1 (9.1%)	3 (5.9%)
Vascular pathologies	1 (6.7%)	4 (4.2%)	1 (9.1%)	3 (5.9%)
Severe anemia	1 (6.7%)	4 (4.2%)	1 (9.1%)	1 (2.0%)
Co-infections*	1 (6.7%)	2 (2.1%)	1 (9.1%)	2 (3.9%)
Maternal Zika infection – no (%)				
Symptomatic	3 (20.0%)	22 (22.9%)	2 (18.2%)	10 (19.6%)
Asymptomatic	12 (80.0%)	74 (77.1%)	9 (81.8%)	41 (80.4%)
Trimester of Maternal Zika infection – no. (%)				
T1 infection	6 (40.0%)	21 (21.9%)	4 (36.4%)	15 (29.4%)
T2 infection	5 (33.3%)	34 (35.4%)	4 (36.4%)	17 (33.3%)
T3 infection	2 (13.3%)	22 (22.9%)	2 (18.2%)	10 (19.6%)
Unknown	2 (13.3%)	19 (19.8%)	1 (9.1%)	9 (17.6%)
Dichorionic twins	0 (0.0%)	5 (5.2%)	0 (0.0%)	3 (5.9%)
Fetus gender – no. (%)				
Female	7 (46.7%)	55 (57.3%)	5 (45.4%)	33 (64.7%)
Male	8 (53.3%)	41 (42.7%)	6 (54.6%)	23 (35.3%)
Term at birth (weeks' gestation) - median (min-max)	39 (32-41)	39 (36-41)	39 (37-41)	39 (36-41)
Premature birth <37wg	1 (6.7%)	6 (6.3%)	0 (0.0%)	3 (5.9%)
Mode of delivery – no. (%)				
Normal	13 (86.7%)	86 (89.6%)	10 (90.9%)	44 (86.3%)
C-section	2 (13.3%)	10 (10.4%)	1 (9.1%)	7 (13.7%)
Neonatal adaptation				
Abnormal Apgar score (<6 at 5 min) – no (%)	2 (13.3%)	11 (11.5%)	2 (18.2%)	5 (9.8%)
Abnormal Lactate level (> 4.5) – no (%)	2 (13.3%)	13 (13.5%)	1 (9.1%)	7 (13.7%)
Structural brain anomalies	5 (33.3%)	3 (3.1%)	4 (36.4%)	2 (3.9%)
Neurological symptoms at birth	4 (26.7%)	1 (1.0%)	4 (36.4%)	1 (2.0%)
Neurologic impairments during infancy	NA	NA	4 (36.4%)	3 (5.9%)
Neurosensory alterations	NA	NA	4 (36.4%)	1 (2.0%)

$^{\pi}$ including multiple maternal co-morbidities

* one primary cytomegalovirus infection in the infected group; one primary CMV and one primary

Alcool consumption was defined as ongoing consumption during the pregnancy after its diagnosis

Current smoking was defined as ongoing smoking during pregnancy after its diagnosis

Trimester of maternal Zika infection was estimated based on symptoms onset, or on laboratory results (asymptomatic)

Supplementary Table 3: Effect of maternal Zika infection diagnosed in the first trimester on main outcomes

Stratified analyses are presented according to Mantel-Haenszel methods. "} p-value" refers to the test of homogeneity between RR associated with confirmed congenital infection after maternal infection in the first trimester and RR associated with confirmed congenital infection after maternal infection in the second or third trimesters.

Main outcomes	Confirmed congenital infections	Negative neonatal testing	RR [95%CI]	p
Neonatal and early infantile adverse outcomes[1]	8/18 (44.4%)	4/111 (3.6%)	12.3 [4.1-36.8]	<0.001
- maternal infection in the 1st trimester	6/7 (85.7%)	2/27 (7.4%)	11.6 [2.9-45.2]	} 0.7200
- maternal infection in the 2nd or 3rd trimester	2/11 (18.2%)	2/84 (2.4%)	7.6 [1.2-48.9]	
Adverse outcomes at 2 years of life[2]	6/15 (40.0%)	5/96 (5.2%)	7.7 [2.7-22.1]	<0.001
- maternal infection in the 1st trimester	3/6 (50.0%)	2/20 (10.0%)	5.0 [1.1-23.3]	} 0.6239
- maternal infection in the 2nd or 3rd trimester	3/9 (33.3%)	3/76 (3.9%)	8.4 [2.0-35.8]	
Referral for suspicion of neurodevelopment <-2SD in at least one domain at 3 years of life[3]	7/11 (63.6%)	7/51 (13.7%)	4.6 [2.0-10.5]	<0.001
- maternal infection in the 1st trimester	3/4 (75.0%)	3/15 (20.0%)	3.8 [1.2-12.0]	} 0.7010
- maternal infection in the 2nd or 3rd trimester	4/7 (57.1%)	4/36 (11.1%)	5.1 [1.7-15.8]	

[1] 129 infants evaluated from birth to 2 months of life

[2] 111 children evaluated up to 2 years of life

[3] 62 ichildren evaluated at 3 years of life using the Child Development Assessment Scale

Stratified analysis presented according to Mantel-Haenszel methods, } *p* -value reffered to the test of homogeneity between the two strata
