# Supplementary material for: Association between confirmed congenital Zika infection at birth and outcomes up to 3 years of life
Source: Nat Commun. 2021 Jun 1;12:3270. doi: 10.1038/s41467-021-23468-3 (PMC8169933; doi:10.1038/s41467-021-23468-3)
Supplement: Supplementary file 4 — Reporting Summary [file 41467_2021_23468_MOESM4_ESM.pdf]

nature research

Corresponding author(s): Léo Pomar

Last updated by author(s): Apr 21, 2021

Reporting Summary

Nature Research wishes to improve the reproducibility of the work that we publish. This form provides structure for consistency and transparency in reporting. For further information on Nature Research policies, see our Editorial Policies and the Editorial Policy Checklist.

Statistics

For all statistical analyses, confirm that the following items are present in the figure legend, table legend, main text, or Methods section.

n/a	Confirmed	
☐	☒	The exact sample size (n) for each experimental group/condition, given as a discrete number and unit of measurement
☐	☒	A statement on whether measurements were taken from distinct samples or whether the same sample was measured repeatedly
☐	☒	The statistical test(s) used AND whether they are one- or two-sided<br>*Only common tests should be described solely by name; describe more complex techniques in the Methods section.*
☐	☒	A description of all covariates tested
☐	☒	A description of any assumptions or corrections, such as tests of normality and adjustment for multiple comparisons
☐	☒	A full description of the statistical parameters including central tendency (e.g. means) or other basic estimates (e.g. regression coefficient) AND variation (e.g. standard deviation) or associated estimates of uncertainty (e.g. confidence intervals)
☐	☒	For null hypothesis testing, the test statistic (e.g. F, t, r) with confidence intervals, effect sizes, degrees of freedom and P value noted<br>*Give P values as exact values whenever suitable.*
☒	☐	For Bayesian analysis, information on the choice of priors and Markov chain Monte Carlo settings
☒	☐	For hierarchical and complex designs, identification of the appropriate level for tests and full reporting of outcomes
☒	☐	Estimates of effect sizes (e.g. Cohen's d, Pearson's r), indicating how they were calculated

Our web collection on statistics for biologists contains articles on many of the points above.

Software and code

Policy information about availability of computer code

Data collection	Excel 2016 software (Microsoft, WA, USA)
Data analysis	Stata15 (Stata Corporation, College Station, TX, USA).

For manuscripts utilizing custom algorithms or software that are central to the research but not yet described in published literature, software must be made available to editors and reviewers. We strongly encourage code deposition in a community repository (e.g. GitHub). See the Nature Research guidelines for submitting code & software for further information.

Data

Policy information about availability of data

All manuscripts must include a data availability statement. This statement should provide the following information, where applicable:

- Accession codes, unique identifiers, or web links for publicly available datasets
- A list of figures that have associated raw data
- A description of any restrictions on data availability

Source data that underlie the results are provided with this paper. Other individual participant data and codes will be shared with researchers who provide a methodologically sound proposal for multi-centric study, particularly individual participant data meta-analysis. Proposals should be directed to leo.pomar@chuv.ch.

Field-specific reporting

Life sciences study design

All studies must disclose on these points even when the disclosure is negative.

Sample size	No sample-size calculation was performed, as we recruited all newborns from Zika-infected mothers followed at the pediatric clinic of the CHOG after parental consent.
Data exclusions	Asymptomatic neonates from mothers living along the Maroni river, outside of the Saint-Laurent du Maroni area, were discharged with their mother after day 3-5 post-partum and were followed in the nearest primary healthcare center, and only came back to the CHOG in case of emergency or need for advanced care. Thus, these infants were not included in this cohort.
Replication	Findings of this observationnal study were not reproduced, as they were based on the exposure to the 2016-Zika outbreak.
Randomization	We defined a laboratory confirmed congenital ZIKV infection either by positive RT-PCR from at least one fetal/neonatal sample (amniotic fluid, cerebrospinal fluid, urine, blood) or identification of specific IgM in neonatal blood or in cerebrospinal fluid. Neonates from ZIKV-infected mother without a laboratory confirmed congenital ZIKV infection were classified as controls. Baseline characteristics were compared between children with a confirmed congenital infection at birth and controls using standardized differences. Baseline characteristics were considered unbalanced if standardized differences were >0.1. Unbalanced baseline characteristics were controlled as potential confounders or effect modifiers for main outcomes using generalized linear multivariable models.
Blinding	The investigators were blinded for clinical data collection at birth, as the results for congenital Zika infection testing were available generally after post-partum discharge. For the evaluation at three years of life, using the Child Development Assessment Scale, investigators were also blinded for the status of the children. Investigators were not blinded for data analysis, as the data were dichotomized for the analysis of baseline characteristics and outcomes.

Reporting for specific materials, systems and methods

We require information from authors about some types of materials, experimental systems and methods used in many studies. Here, indicate whether each material, system or method listed is relevant to your study. If you are not sure if a list item applies to your research, read the appropriate section before selecting a response.

Materials & experimental systems

n/a	Involved in the study	
☐	☒	Antibodies
☒	☐	Eukaryotic cell lines
☒	☐	Palaeontology and archaeology
☒	☐	Animals and other organisms
☐	☒	Human research participants
☒	☐	Clinical data
☒	☐	Dual use research of concern

Methods

n/a	Involved in the study	
☒	☐	ChIP-seq
☒	☐	Flow cytometry
☒	☐	MRI-based neuroimaging

Antibodies

Antibodies used	In-house MACELISA Zika assay (French Guiana National Reference Centre for arboviruses).
Validation	In adults, its sensitivity, when correlated with PCR results, varying between 87% for serum samples collected between five and 20 days from symptom onset to 98% for those collected after seven days. Specificity varies depending on the presence of co-infections with other arboviruses, reaching 80% in negative patients, but dropping in the case of co-infections (Flamand C et al. Euro Surveill2017;22. doi:10.2807/1560-7917.ES.2017.22.44.17-00102). Serological cross reactions with other Flaviviridae were expected to be minimal, as circulation of Dengue virus has been low in French Guiana since 2014 and no significant circulation of other Flaviviridae has been seen.

Human research participants

Policy information about studies involving human research participants

Population characteristics	Maternal characteristics: median age at birth was 26 years, a majority had a low or moderate socio-economic status (34.1% and 41.9%, respectively); 10.1% and 5.4% of them were exposed to alcohol and lead poisoning during pregnancy, respectively. Maternal Zika infection was diagnosed in the 1st trimester in 26.4% of cases. The rate of dichorionic twin pregnancy was 6.2% in this cohort.<br>Neonatal characteristics: Median gestational age at birth was 39 weeks' gestation, with 5.4% of prematuriy (<37wg). The majority of them (85.3%) was born from natural delivery and presented a good adaptation at birth (Apgar score >6 at 5min in 88.4% of cases).
Recruitment	All newborns from Zika-infected mothers followed at the pediatric clinic of the Centre Hospitalier de l'Ouest Guyannais (CHOG) were recruited, after parental written consent. Newborns initially followed elsewhere and referred to the CHOG for advanced cares were not included he cohort, to avoid selection biases.
Ethics oversight	The study protocol was approved by the institutional review board of the Centre Hospitalier de l'Ouest Guyannais (CHOG).

Note that full information on the approval of the study protocol must also be provided in the manuscript.
